# Reverse Mutations in Pigmentation Induced by Sodium Azide in the IR64 Rice Variety

**DOI:** 10.3390/cimb46120795

**Published:** 2024-11-22

**Authors:** Hsian-Jun Chen, Anuchart Sawasdee, Yu-Ling Lin, Min-Yu Chiang, Hsin-Yi Chang, Wen-Hsiung Li, Chang-Sheng Wang

**Affiliations:** 1 Department of Agronomy, National Chung Hsing University, Taichung 402, Taiwan; lily7232001@hotmail.com (H.-J.C.); anucharts@smail.nchu.edu.tw (A.S.); eikyo1031@email.nchu.edu.tw (Y.-L.L.); dinosaurur@smail.nchu.edu.tw (M.-Y.C.); peppermint33333@gmail.com (H.-Y.C.); 2 Biodiversity Research Center, Academia Sinica, Taipei 115, Taiwan; 3 Department of Ecology and Evolution, University of Chicago, Chicago, IL 60637, USA

**Keywords:** *Oryza sativa*, sodium azide, mutagenesis, pigmentation, reverse evolution, retrotransposon

## Abstract

Pigmentation in rice is due mainly to the accumulation of anthocyanins. Five color mutant lines, AZ1701, AZ1702, AZ1711, AZ1714, and AZ1715, derived from the sodium azide mutagenesis on the non-pigmented IR64 variety, were applied to study inheritance modes and genes for pigmentation. The mutant line AZ1711, when crossed with IR64, displays pigmentation in various tissues, exhibiting a 3:1 pigmented to non-pigmented ratio in the F_2_ progeny, indicating a single dominant locus controlling pigmentation. Eighty-four simple sequence repeat (SSR) markers were applied to map the pigment gene using 92 F_2_ individuals. RM6773, RM5754, RM253, and RM2615 markers are found to be linked to the color phenotype. RM253 explains 78% of the phenotypic variation, implying linkage to the pigmentation gene(s). Three candidate genes, *OsC1* (*MYB*), *bHLH*, and *3GT*, as anthocyanin biosynthesis-related genes, were identified within a 0.83 Mb region tightly linked to RM253. PCR cloning and sequencing revealed 10 bp and 72 bp insertions in the *OsC1* and *3GT* genes, respectively, restoring pigmentation as in wild rice. The 72 bp insertion is highly homologous to a sequence of *Ty1*-*Copia* retrotransposon and shows a particular secondary structure, suggesting that it was derived from the transposition of *Ty1*-*Copia* in the IR64 genome.

## 1. Introduction

In rice (*Oryza sativa* L.), most cultivars are green, but in the abundant seed source of rice, red, purple, etc., can be found, which is due to the accumulation of anthocyanins [1]. Rice color mutants can show various degrees of color expression in different organs or tissues at the same or different growth stages [2]. Pigmentation is a morphological marker for variety identification, linkage analysis, and artificial selection. The color expression of anthocyanins is affected by both genotypic and environmental effects. Previous reports indicated that the accumulation of anthocyanin compounds is determined by the expression of multiple enzymes in metabolic pathways that synthesize anthocyanins [3,4]. The expression of these structural genes is regulated by their cognate transcription factors (TFs), which control the content, pattern, accumulation site, and expression period of anthocyanin compounds [5,6,7]. The expression control of anthocyanins in different plant parts is also influenced by the genetic regulation of different tissue-specific genes or the interaction between genes [1], and even by complex regulatory mechanisms such as post-transcriptional modification and epigenetics [8]. Therefore, the genetic mechanism of anthocyanin production and regulation remains a significant focus of the research topic.

Sodium azide (NaN_3_) is a well-known common bactericide, pesticide, industrial nitrogen gas generator, and inhibitor of the heavy metal enzyme. Although sodium azide is inefficient in Arabidopsis, it has been reported as a potent mutagen in several crops like maize, barley, rice, and soybean [9,10]. Wyss et al. (1948) were the first to discover that the application of sodium azide caused the accumulation of hydrogen peroxide and inhibition of the reactions of catalase and peroxidase and might induce mutation indirectly [11]. Therefore, sodium azide mutagenesis is an azide compound that interacts with DNA in the nucleus under the acidic condition [12,13]. The frequency of point mutation is high, while that of chromosome breakage is lower in sodium azide-induced mutations than in other mutagens [14]. However, factors affecting sodium azide’s mutagenicity include dosage, treatment time, pH, temperature, oxygen concentration, etc. [12,15].

Transposable elements (TEs) are movable DNA elements in the genome of living cells. Sodium azide treatment under specific conditions stresses the DNA during mutagenesis, similar to the mutations in maize by a TE. TEs are classified into two groups: the RNA type TEs (retrotransposons), which transpose in a manner of copy and paste in the genome, and the DNA type TEs, which transpose in a cut and paste manner. Retrotransposons are inactive under normal conditions but can be activated by stress [16]. Hirochika et al. (1996) first reported that the copy number of *Tos17*, a rice retrotransposon, increased during rice tissue culture; as the duration was prolonged, the abnormal phenotypes increased [17,18].

NaN_3_ has been reported to induce pigmentation in vegetable cowpea (*Vigna unguiculata* (L.) Walp.) [19] and to reactivate LTR retrotransposons in plants such as wheat (*Triticum aestivum* L.) [20]. The retrotransposon has been reported to induce pigmentation in grape (*Vitis labruscana*) [21], sweet orange (*Citrus sinensis* L.) [22], and Harlequin (*Phalaenopsis*) orchids [23]. Although there were many reports about pigmentation and retrotransposons in many crops, a lack of understanding in rice, especially in reverse mutation, the recovery function of pigmentation biosynthesis by the insertion of retrotransposons, still remains [24].

The sodium azide-induced mutant pool of the popular *indica* rice cultivar IR64, called the AZ mutants, comprises several mutants with purple color expressions, and the mutants with color expressions in different plant parts were selected for further study. To unravel the mechanism of sodium azide mutagenesis, this study analyzes the genetics of the mutant line AZ1711 and four other stable mutant lines, AZ1701, AZ1702, AZ1714, and AZ1715, all of which show colors on the leaf sheath, auricle, ligule, and apiculus. In addition, AZ1711 is crossed with wild-type IR64 to study the characteristics and genetics of color variation induced by sodium azide. We identify mutations related to color traits in the pigment mutants and propose that the reverse evolution of anthocyanin pigmentation genes can occur through the retrotransposition of retrotransposons.

## 2. Materials and Methods

### 2.1. Mutant Line Selection

The seeds of the rice (*Oryza sativa* L.) cultivar IR64 were treated with 1, 2, 5, and 10 mM of NaN_3_ solution according to the method of Wang et al., 2002 [25]. Seedlings from these treatments were then transplanted into a paddy field. Since M_1_, plants that exhibited distinct morphological characteristics different from their wild-type IR64 were harvested. Subsequent generations, from M_2_ to M_8_, were developed using the single seed descent (SSD) method until the traits were not segregated, as described in reference [26]. No other purple rice plants were present in the paddy field during the M_1_ to M_8_ generations, ensuring that the observed mutant lines are true mutations and become pure (mutant) lines.

In this study, the five mutant lines AZ1701, AZ1702, AZ1711, AZ1714, and AZ1715 were selected from the AZ mutant pool because they show colors on many plant tissues, including leaf sheath, auricle, ligule, and apiculus. Also, Nipponbare and 93-11 were used as the references for the *japonica* and *indica* types of rice, respectively.

### 2.2. Phenotyping of Tissue Colors

Plant pigmentation was investigated following the methods of the International Rice Research Institute [27]. The pigmentations on tissues were investigated in five mutation lines (AZ1701, AZ1702, AZ1711, AZ1714, and AZ1715). The tissues were judged by the presence or absence of anthocyanin and color at the proper rice growth stage. However, this study only focused on four tissues: leaf sheath, auricle, ligule, and apiculus.

### 2.3. Establishment of Populations for Genetic Analysis and Linkage Mapping

For genetic analysis, the pigmented mutant lines AZ1701, AZ1702, AZ1711, AZ1714, and AZ1715 were crossed to the wild type, IR64. The F_1_ plants were confirmed to be real heterozygous using SSR markers. All progeny were tested by Chi-square (*χ*^2^) to determine the mode of inheritance according to the segregation ratios of anthocyanin and no anthocyanin traits. The mutant line AZ1711 showed stable pigmentation traits and was selected as the target mutant line in this study. The F_2_ population of the AZ1711×IR64 cross was generated. A total of 557 F_2_ individuals were planted for genetic analysis and linkage mapping.

### 2.4. DNA Extraction and Genotyping

To establish a population for pigmentation mapping, 92 individuals were randomly chosen from the 557 F_2_ individuals. The genomic DNA of these 92 F_2_ individuals, the two parents (AZ1711 and IR64), Nipponbare, and 93-11 were extracted by the CTAB method [28] and genotyped by SSR markers [29].

### 2.5. Linkage Mapping and Fine Mapping

A total of 418 SSR markers were applied in our polymorphism screening (Table S1). At the beginning, the polymorphisms between the two parents were detected using 361 SSR markers from the Cornell SSR 2001 genetic map [30] and the IRMI 2003 genetic map [31] that were distributed over the rice genome. Those SSR markers that were not amplified or their PCR products showed dominant inheritance were discarded. Then, 83 polymorphic co-dominant markers were used to analyze the genotype of the 92 F_2_ individuals. The six markers that rejected the ratio of AA:AB:BB as 1:2:1 (*p* < 0.05) were discarded. The linkage mapping was then conducted with 77 polymorphic SSR markers using the R software with an add-on package R/qtl (version: 1.50) [32]. The genetic distance was estimated by the Kosambi mapping function, and the chromosome number of each SSR marker was assigned according to IRGSP-1.0. The simple interval mapping (SIM) model was employed to detect the locus responsible for pigmentation. A significant logarithm of odds (LOD) threshold (*p* < 0.05) was determined using 1000 permutations [33]. The Bayesian credible region method was applied to calculate the 95% confidence intervals. The polymorphic markers within the mapped region were screened with 57 additional SSR markers for fine mapping. The linkage map was redrawn, and the recombinants of markers were evaluated. Moreover, the single marker regression analysis was employed using QTL/AVONA1 in the MapDisto (version: 1.7.0) software [34].

### 2.6. Candidate Gene Approach, Cloning, and Sequence Comparison

The annotation of the genes within the mapped region (from RM5745 to RM2615 on Chr. 6) was downloaded from the Rice Genome Annotation Project (RGAP) (Release 7 of the MSU Rice Genome Annotation, http://rice.uga.edu/, accessed on 2 May 2022) and the Gramene website Indica Genomes (Ensembl Plants release 53, https://plants.ensembl.org/Oryza_indica/Info/Index, accessed on 7 April 2022). According to the literature review, the candidate genes related to anthocyanin biosynthesis were selected to identify the sequence by PCR cloning. The full-length genomic sequences of candidate genes were downloaded from NCBI (https://blast.ncbi.nlm.nih.gov/Blast.cgi, accessed on 13 May 2022) and Gramene (https://www.gramene.org/, accessed on 13 May 2022) websites, including 2 kb of the upstream and downstream to design gene-specific primers by the Vector NTI (version: 10.3.0) software (Invitrogen Co., Waltham, MA, USA) for PCR amplification using ExTaq polymerase (Takara Co., Tokyo, Japan). The PCR-amplified fragments were purified through DNA Clean-Up & Extraction Kit (GMbiolab Co., Ltd., Taichung, Taiwan) and cloned into a pGEM-T-easy vector (Promega Corporation, Madison, WI, USA) for Sanger sequencing and comparisons. Sequences of IR64 and AZ1711 were aligned with those of Nipponbare and 93-11 from the database, and the mutations as SNPs, transitions, transversions, insertions, and deletions were identified and calculated using the Vector NTI software (version: 10.3.0).

### 2.7. Comparison of Sequences and Promoter Prediction

The homologous sequences were obtained by BLASTN from the Ensembl Plants (https://plantsGramene.ensembl.org/index.html, accessed on 17 May 2022), NCBI (https://blast.ncbi.nlm.nih.gov/Blast.cgi, accessed on 17 May 2022), The Rice Genome Annotation Project Database and Resource (http://rice.uga.edu/index.shtml, accessed on 17 May 2022) and ROOTomics (https://rootomics.dna.affrc.go.jp/en/research/IR64, accessed on 17 May 2022). The promoter was predicted using the BDGP Promoter Prediction (https://www.fruitfly.org/seq_tools/promoter.html, accessed on 19 May 2022). The secondary structure of the 72 bp insertional DNA sequence was predicted using the UNAFold web server (http://www.unafold.org/, accessed on 30 May 2022).

## 3. Results

### 3.1. Plant Tissue Pigmentations in Mutants Induced by Sodium Azide

**Figure 1:**
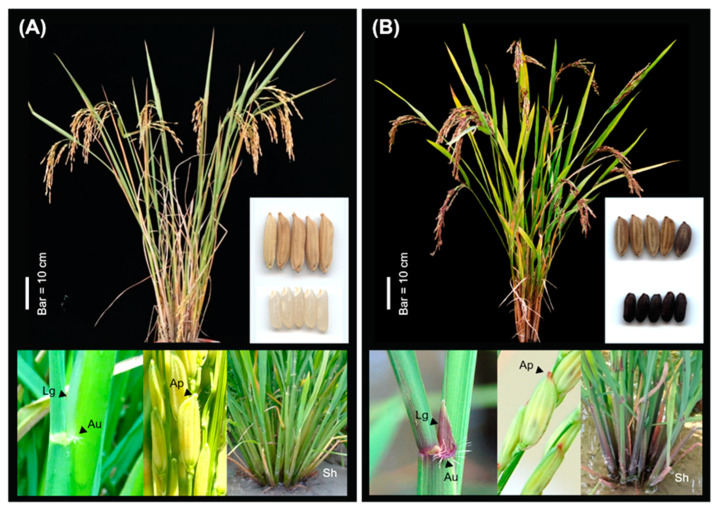
Plant architecture and anthocyanin pigmentation in the plant tissues of (**A**) IR64 and (**B**) its mutant line AZ1711. The tissues auricle (Au), ligule (Lg), apiculus (Ap), and sheath (Sh) are labeled.

There is no pigmentation in the tissues of the wild-type IR64 rice variety. In contrast, the AZ1711 mutant line shows colors in most tissues (Figure 1), suggesting that genes involved in pigment biosynthesis have been mutated. The other four mutant lines under study also show colors in various plant tissues (Figure S1). Indeed, about 230 out of the 1800 lines in the mutation pool of the IR64 variety show colors in their plant tissues. Therefore, color mutations are commonly induced by sodium azide in rice plants.

The color characteristics of AZ1711 are purple leaf sheath, purple ligule, light purple auricle, light purple leaf collar, purple-black stigma, purple-red apiculus, purple leaf margin, purple palea, and purple lemma (Figure 1). The other four mutants under study also show similar pigmentation on the leaf sheath, ligule, auricle, and apiculus (Table 1, Figure S1), indicating that they may contain mutations like AZ1711. In addition, some mutations can be observed, such as the colored hull on the grains of AZ1711, AZ1714, and AZ1715, and on a long awn of AZ1701 (Figure S1). Since various genes or pathways controlling the pigmentation of vegetative tissues and grains (hull and pericarp) have already been reported in rice [1,2,35], we focused on the color mutations on the four tissues that showed a single dominant inheritance (Table 1).

### 3.2. Genetic Mapping and the Candidate Gene Approach

As mentioned above, IR64 exhibits a whole plant green (G) phenotype, while its mutant lines AZ1701, AZ1702, AZ1711, AZ1714, and AZ1715 display various colors in some plant tissues (Figure 1 and Figure S1). To simplify the description, we classify the wild type IR64 as non-pigmented or green and the color mutants as pigmented (including all colors except green). All the F_1_ plants of the five lines crossed with IR64 showed pigmentations in the four tissues under study, indicating that the pigment biosynthetic pathway has been activated even in the heterozygous genotypes, and the pigmentation on mutants is expressed dominantly. The four targeted tissues (leaf sheath, auricle, ligule, and apiculus) of the 557 F_2_ individuals were segregated in the ratio of three purples (pigment)–one green (non-pigment) (*χ*^2^ = 2.45, 2.77, 3.10, and 2.77 for the above 4 tissues, respectively). The results suggest that all four tissues are conditioned by the same single dominant mutated locus or a group of linked genes resulting in the pigmentation of these four tissues (Table 1).

**Figure 2:**
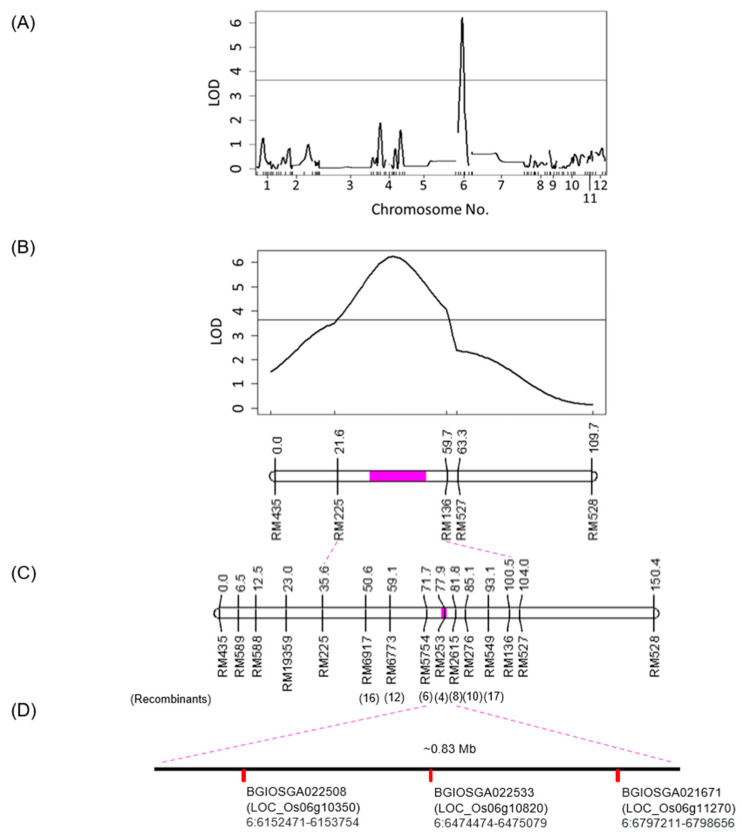
A genetic and physical map of the locus that controls leaf sheath, auricle, ligule, and apiculus pigmentation in the AZ1711×IR64 cross. Genome-wide LOD plots of QTL analyses in different regression models by the Haley–Knott regression method. (**A**) LOD plot from the whole-genome scan. (**B**) LOD plot of Chr. 6 only. The *X*-axis indicates the relative position on the linkage map, and the *Y*-axis represents the LOD score. The solid line indicates the LOD significance threshold of 3.61. (**C**) Fine mapping and the locus responsible for the pigmentation mapped on Chr. 6 within a 0.83 Mb region. A filled pink area shows the 95% Bayesian credible interval and the number of recombinants shown below the markers. (**D**) The three candidate genes were obtained from Gramene (release 64) that may be related to the pigmentation in AZ1711.

For the genetic mapping of the gene that controls the pigmentation of the four tissues, 92 F_2_ individuals from the 557 F_2_ population of AZ1711×IR64 cross (Table 1) were subjected to genotyping using SSR markers. A total of 361 SSR markers selected from the Cornell SSR 2001 genetic map [30] and the IRMI 2003 genetic map [31] (Table S1) were applied to screen the polymorphism between IR64 and AZ1711, and 111 of them were examined as co-dominant markers. In 83 of the 111 polymorphic markers, the genotypes could be determined more precisely and were tested on the 92 F_2_ individuals. Only six SSR markers did not show the 1:2:1 ratio (*p*-value < 0.05), and were excluded in the further analysis. Therefore, 77 polymorphic SSR markers were used to construct a linkage map by R/qtl (Figure S2). After the R/qtl analysis, a locus responsible for the pigmentation was mapped between the RM225 and RM136 markers, spanning in a ~5.3 Mb region on Chr. 6, which explains 26.8% (LOD = 6.23) of the phenotypic variation (Figure 2A,B).

To increase the marker density on Chr. 6 for fine gene mapping, another 57 SSR markers within the mapped region were applied to screen for polymorphism. Seven polymorphic SSR markers were found, including RM6917, RM6773, RM5754, RM253, RM2615, RM276, and RM549. These markers were included in the R/qtl analysis for fine mapping, and the mapped region was narrowed down to ~0.83 Mb between RM5754 and RM2615, and the RM253 marker showed the lowest recombination frequency (Figure 2C). The phenotypic variance explained by this region was raised to 78.4% (LOD = 30.66).

Using QTL/AVONA1 in the MapDisto software to perform the single-marker analysis identified the four markers, RM6773, RM5754, RM253, and RM2615, by the *t*-test (*p*-values = 1.41 × 10^−11^, 2.11 × 10^−11^, 7.24 × 10^−31^, and 2.78 × 10^−18^, respectively) (Table 2). Among these four markers, RM253 showed the *R*^2^ value, additives (A) effect, and dominant (D) effect of 0.78, −0.45, and 0.46, respectively, and the ratio |D/A| = 1.03. The *R*^2^ of the flanking markers RM5754 and RM2615 are 0.59 and 0.43, respectively, indicating that these markers are highly correlated or linked with the genes that control the pigmentation of the four tissues. Therefore, the phenotype of the F_2_ plants inferred from these markers suggests that our target gene(s) may be located between the RM5745 and RM2615 markers (Figure 2B).

According to the fine-mapped interval from RM5745 to RM2615 on Chr. 6 (Figure 2C), the candidate gene sequences were searched and retrieved from the Gramene website Indica Genomes (Ensembl Plants release 53, Oryza_indica–Ensembl Genomes 53, accessed on 7 April 2022) and the Rice Genome Annotation Project (RGAP, Release 7 of the MSU Rice Genome Annotation, http://rice.uga.edu/, accessed on 2 May 2022). Among them, the *indica* rice 93-11 contains 93 ORFs with gene structure and the *japonica* rice contains 111 annotated genes (Table S2). We applied the candidate gene approach to screen the possible genes according to the gene functional annotation and found three genes related to anthocyanin biosynthesis in this region (Figure 2D, Table 3).

The first gene, *OsC1*, whose MSU ID is LOC_Os06g10350, was annotated as a TF in the MYB family and was named anthocyanin regulatory C1 protein [36]. The second gene, *bHLH* (LOC_Os06g10820), was annotated as a helix-loop-helix (bHLH) DNA-binding domain-containing protein and is also a TF in anthocyanin biosynthesis [37]. The third gene, *3GT* (LOC_Os06g11270), was annotated as anthocyanidin 3-O-glucosyltransferase (3GT) [38]. Therefore, these genes of AZ1711 and IR64 were cloned and sequenced to examine their sequences.

### 3.3. Comparison of OsC1, bHLH, and 3GT Sequences in Mutant Lines and the Wild Type

**Figure 3:**
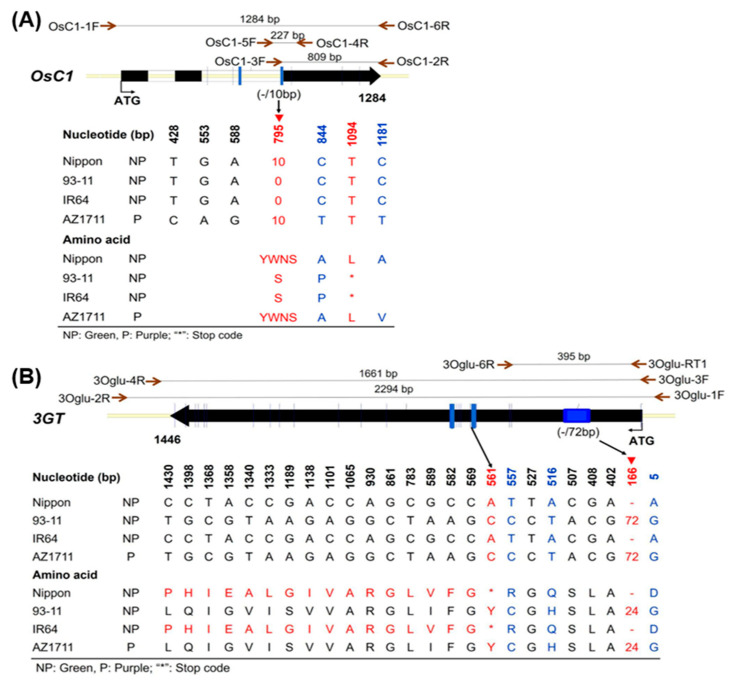
Mutation of the candidate genes in the mapped region between RM253 and RM2615 markers defined from Nipponbare, 93-11, and wild type IR64 and its anthocyanidin mutant line AZ1711. (**A**) The SNPs and amino acids are labeled under the *OsC1* gene. (**B**) The SNPs and amino acids are listed under the *3GT* gene, and primers for PCR are labeled above genes. Nucleotides in blue represent nonsynonymous SNPs, those in black indicate synonymous SNPs, and the red dash indicates In/Del mutations. Red stars represent the stop codon.

The first mapped candidate gene, *OsC1*, is found in Nipponbare and 93-11 at the Gramene database IRGSP-1.0 and ASM465v1. It encodes a MYB-like TF with three exons and a coding sequence of 1284 bp. The sequence from 2 kb upstream of the 5’ end to 2 kb downstream of the 3’ end of this gene was downloaded and imported into the Vector NTI software, and two primers OsC1-1F and OsC1-6R (Table S3) flanking the coding region were designed for gene cloning using PCR (Figure 3A). The *OsC1* genes (~1.3 kb) in the wild-type IR64 and the mutant AZ1711 were amplified by PCR, purified, cloned, and sequenced by Sanger sequencing. Sequence comparison found a 10 bp insertion in the third exon of *OsC1* in AZ1711. The translation showed that the 10 bp deletion in the wild-type IR64 caused an early stop at the 1103 bp position and shortened the peptide from 210 to 107 amino acids (Figure 3A and Figure S3). The 10 bp insertion started at the 795 bp of the *OsC1* in AZ1711 and recovered the full-length functional protein like the wild rice *O. rufipogon* (Figure 3A and Figure S3, Table S4).

The second candidate gene, LOC_Os06g10820, is annotated in Gramene and encodes a Myc-like TF with a bHLH domain structure. The 93-11 variety is 606 bp long, contains one exon, and encodes a protein of 201 amino acids. Two primers, bHLH-3F and bHLH-4R, were designed for PCR-cloning the sequence of 2032 bp from ~1 kb upstream to ~1 kb downstream of the gene. No difference was found between IR64 and AZ1711, indicating that the *bHLH* gene does not influence the pigmentation of AZ1711.

The third candidate gene, LOC_Os06g11270, is the *3GT* gene; the coding region has 1446 bp in the 93-11 variety, with only one exon, and encodes a peptide of 482 amino acids. Two primers, 3Oglu-3Fand 3Oglu-4R were designed (Table S3), and a 1661 bp fragment was amplified by PCR. The sequence comparison revealed the insertion of a 72 bp fragment (at 166 bp) in AZ1711, which is not found in its wild type of IR64 (Figure 3B and Figure S4). In addition to the 72 bp insertion in 3*GT*, 24 base changes were found in AZ1711, including 13 transition mutations (Ts) and 11 transversion (Tv) mutations (Figure 3B). The 72 bp deletion in IR64 would result in the missing of 24 amino acids in the 3GT protein. Moreover, the 24 base changes would result in one nonsense (at 561), 11 missense (at 5, 516, 557, 589, 1138, 1189, 1333, 1340, 1358, 1398, and 1430), and 12 synonymous (at 402, 408, 507, 527, 569, 582, 783, 861, 930, 1065, 1101, and 1368) changes, which would disrupt the function of the 3GT protein. Furthermore, the nucleotide at the 561 bp position is A (adenine) in the non-pigmented wild type, which becomes a stop codon TAA and terminates the translation prematurely, producing a peptide of only 187 amino acids with no function in pigment biosynthesis. Interestingly, the A561C (TAA → TAC) mutation in the AZ1711 mutant changes a stop codon to tyrosine (Y), providing a functional 3GT protein that produces pigmentations (Figure 3B and Figure S4).

According to our study, two (*OsC1* and *3GT*) of the candidate genes in the mapped region result in two gain-of-function mutations in AZ1711, each of which recovered pigmentation.

### 3.4. Identification of Insertion Fragments in the Mutated Genes of Pigmented Lines

**Figure 4:**
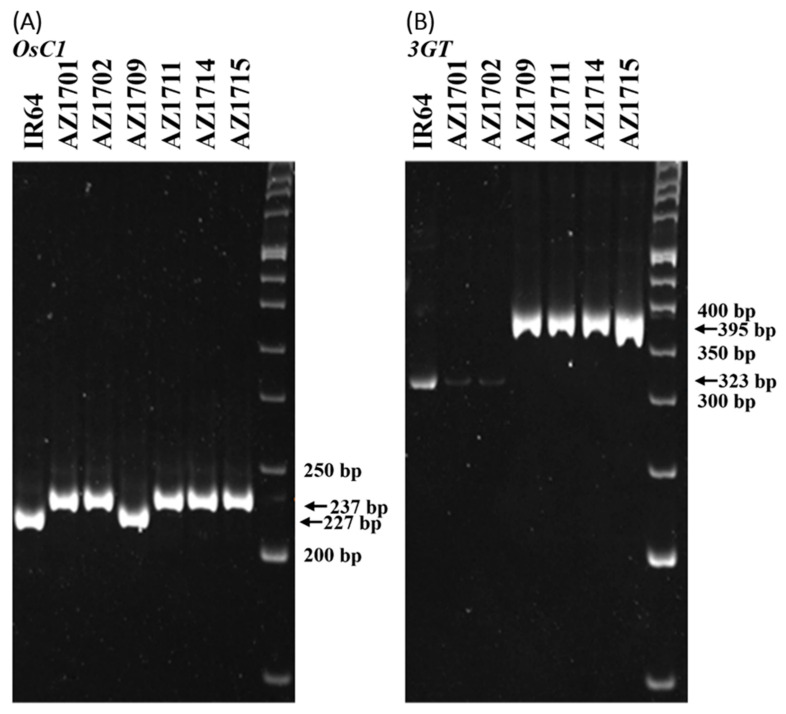
Polymorphism of *OsC1* and *3GT* gene-specific fragments amplified in the pigmented mutant lines. (**A**) The gDNA fragments of the *OsC1* gene amplified by the OsC1-5F and OsC1-4R primers in Nipponbare, 93-11, IR64, AZ1701, AZ1701.1, AZ1709, AZ1711, AZ1714, and AZ1715, respectively. (**B**) The gDNA fragments of the *3GT* gene by the 3Oglu-RT1 and 3Oglu-6R primers in Nipponbare, 93-11, IR64, AZ1701, AZ1701.1, AZ1709, AZ1711, AZ1714, and AZ1715, respectively.

Gene-specific primers are designed to amplify the inserted fragments from both the *OsC1* and *3GT* genes to confirm the mutations. For *OsC1*, fragments of 227 bp and 237 bp (+10 bp) are expected to be amplified from this gene in IR64 and AZ1711, respectively, by the OsC1-5F and OsC1-4R primers. Similarly, fragments of 323 bp and 395 bp (+72 bp) are expected to be amplified from the *3GT* gene in IR64 and AZ1711, respectively, by the 3Oglu-RT1 and 3Oglu-6R primers (Figure 4). The gene-specific primers are also applied to genotype the four other color mutant lines under study. The data show that the 10 bp insertion is also found in the *OsC1* gene of AZ1701, AZ1702, AZ1711, AZ1714, and AZ1715, which have colors in the leaf sheath, auricle, ligule, and apiculus, but it is not found in their wild type IR64 (Figure 4A). In the *3GT* gene, the 72 bp insertion was found in AZ1711, AZ1714, and AZ1715 but not in IR64, AZ1701, or AZ1702 (Figure 4B). These results indicate that *OsC1* is indeed related to the pigmentation of the five mutants, but *3GT* may not be responsible for the color expression in AZ1701 and AZ1702. The sequence data from the Gramene database show that the 10 bp insertion in *OsC1* can be found in Nipponbare but not in 93-11, while the 72 bp insertion in the *3GT* gene cannot be found in Nipponbare but is found in 93-11 (Figure 3, Figures S3 and S4).

**Figure 5:**
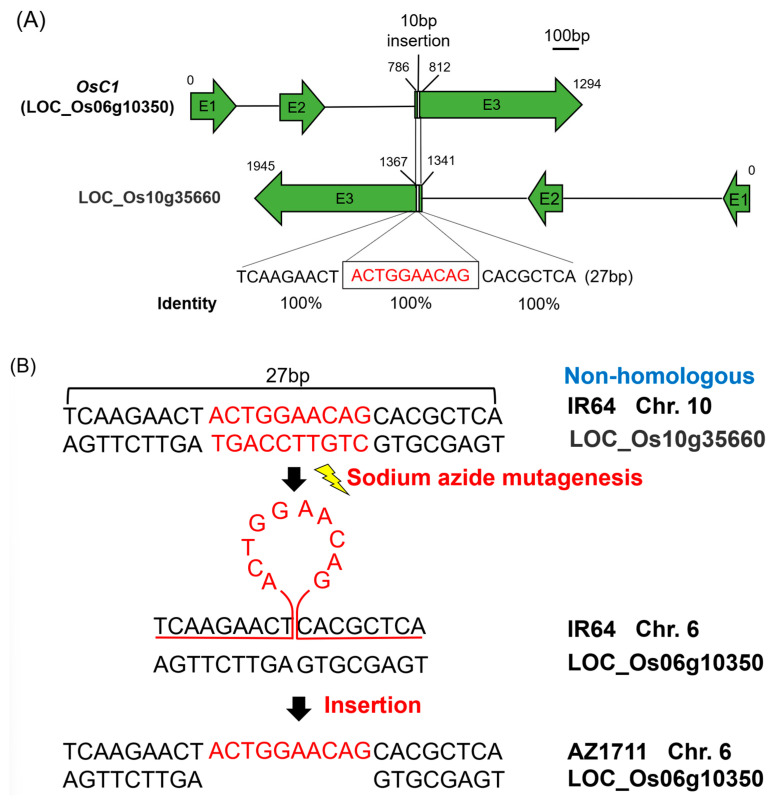
Comparison of the homologous sequences of *OsC1* in the genome of IR64. (**A**) A 10 bp insertion of *OsC1* is identical to the R2R3 domain of LOC_Os10g35660 in AZ1711. (**B**) The 10 bp insertion might be popped out during DNA replication or repair from the 27 bp of LOC_Os10g35660 (*OsC1*) on Chr. 6 and the *MYB* gene on Chr. 10 during NaN_3_ mutagenesis.

The next challenge is to identify the origins of the two insertional fragments and the mechanisms underlying the two mutations. The insertion of a DNA fragment into a chromosome requires that the insertion site in the chromosome has two flanking sequences that are highly homologous to the two end sequences of the fragment. As mentioned above, the *OsC1* gene in the color mutant AZ1711 was inserted by a 10 bp fragment. To understand how AZ1711 gained the 10 bp insertion, the sequences that include the 10 bp sequence and its 500 bp up and downstream sequences were used to BLAST the NCBI and Ensembl Plants databases. The results showed that Chr. 3, 4, 9, and 10 are aligned with 100% identity to the 10 bp insertion in AZ1711, but the 27 bp fragment in Chr. 10 is the only longer fragment with 100% identity. The 27 bp segment in AZ1711, including the 10 bp insertion fragment, is entirely identical to the reversed segment located in Chr. 10 (LOC_Os10g35660) in *Oryza sativa* ssp. *japonica* (Nipponbare) and *O. sativa* ssp. *indica* (93-11) (Figure 5). The orthologous fragment on Chr. 10 also encodes an MYB TF-like gene; however, its function has not yet been defined in rice.

**Figure 6:**
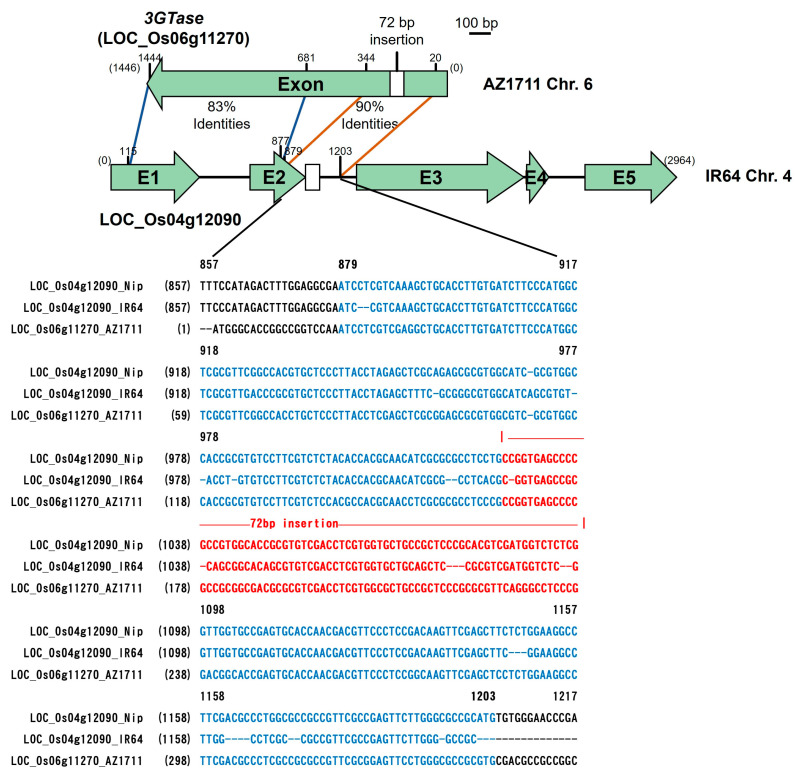
Comparison of the homologous sequences of *3GT* in the AZ1711 genome. The *3GT* (LOC_Os06g11270) and the LOC_Os04g12090 genes have homologous sequences. Two sequence homologs of the *Ty1-Copia* retrotransposon (RT) (LOC_Os04g12090) with 90% and 83% identities with Nipponbare and AZ1711, respectively. The blue-colored sequence represents the region that showed 90% and 83% identities. The red-colored sequence represents the 72 bp insertion. The white boxes represent the 72 bp insertion of AZ1711 and the green boxes represent exons (E1–E5). The non-pigment of Nipponbare is due to the deletion of the 72 bp fragment.

**Figure 7:**
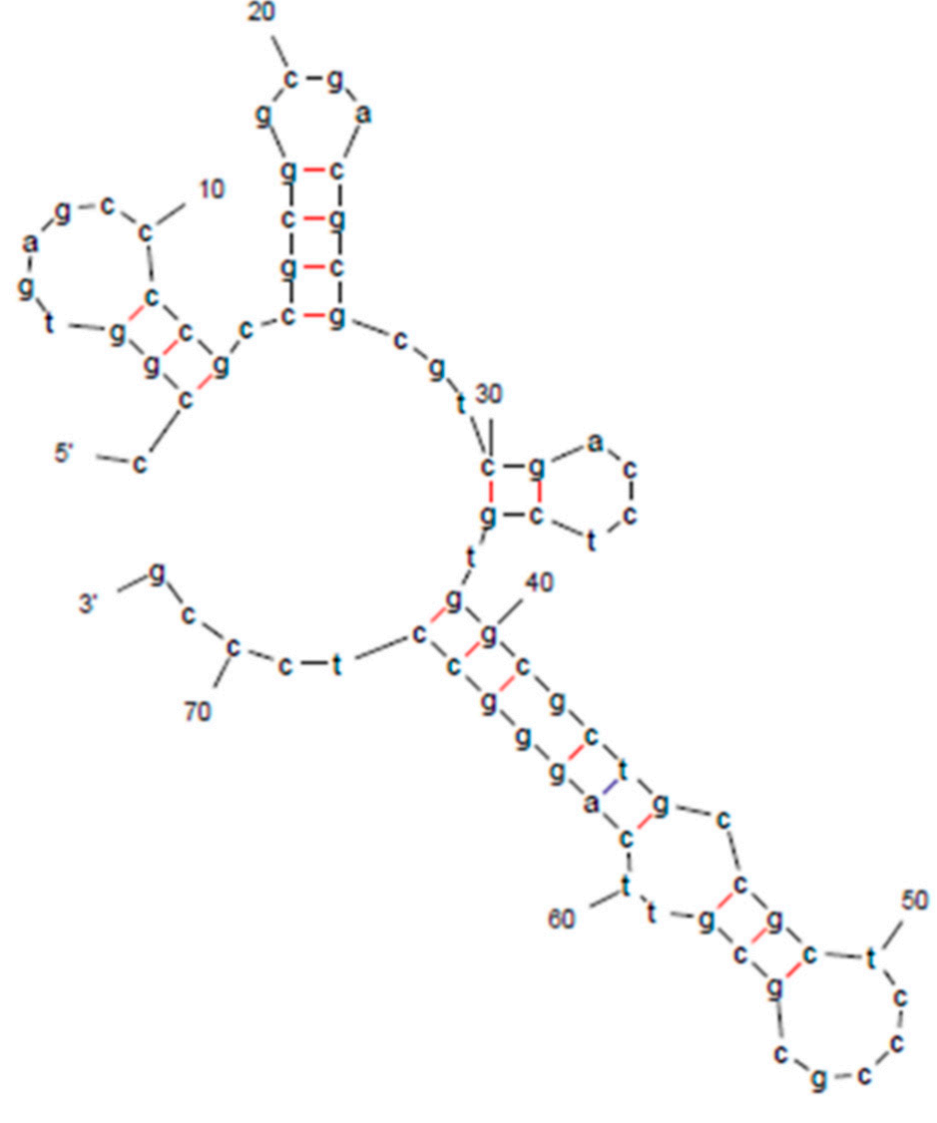
The predicted secondary structure of the 72 bp insertional fragment in the *3GT* of the AZ1711 line contains four hairpin loops (at ~10th, 20th, 35th, and 50th nucleotide positions), a multi-loop (5′–3′), and an internal loop (at ~60th nucleotide position).

We also found a sequence on Chr. 10 homologous to the 72 bp fragment of the *3GT* in AZ1711 with high nucleotide identity (94.2%) in the gene annotated as a glycosyltransferase family gene in the Nipponbare genome (Figure 6). However, other homologous sequences (90% identity) on Chr. 4 also were found to be annotated as a putatively expressed *Ty1-Copia* retrotransposon (Figure 6). Therefore, the full-length sequence of the retrotransposon (LOC_Os04g12090) in the Nipponbare is compared with the full-length *3GT* sequence (LOC_Os06g11270) of the color mutant AZ1711. The comparison revealed that the sequences of the *3GT* gene have an identity high enough to pair with the sequences on Chr. 4 and/or Chr. 6 in the rice genome of 93-11 and many others with color. The full-length genes share only partial sequence identities, but the identities near the inserted sequence are as high as 90% (Figure 6). Thus, the 72 bp insertional sequence in the color mutant AZ1711 may be related to the retrotransposon. Further sequence and structure analyses found that the 72 bp insertional fragment, similar to part of *Copia*-LTR retrotransposon, has unique secondary structures that are not commonly found in genes. Still, they can provide an easy-recognition target during DNA replication and/or repair after mutagenesis (Figure 7).

## 4. Discussion

This study, which used a series of pure-line mutants developed from the IR64 rice variety by sodium azide mutagenesis, provides several new findings in sodium-azide-induced mutations in rice:

### 4.1. Identification of a Specific Region for Pigmentation in Rice

According to our genetic analysis, the pigmentations in the leaf sheath, auricle, ligule, and apiculus of the pigment mutant AZ1711 are controlled by a single dominant locus (Table 1). Therefore, 92 F_2_ individuals from the cross AZ1711×IR64 were sufficient for constructing a linkage map [39,40]. The differences between the two parental genomes were screened using 361 SSR markers covering the rice genome [41]. The results showed that 38.5% of SSR markers could differentiate between IR64 and AZ1711 (Table S1). In addition, while a previous study reported that only point mutations were induced by sodium azide in barley [42], the present study found that insertions and deletions can also be induced by sodium azide.

The pigmentation locus was first mapped between RM225 and RM136, spanning a 5.3 Mb region on Chr. 6 (Figure 2A,B), then, narrowed down to ~0.83 Mb between RM5754 and RM2615 (Figure 2C), and finally, found to be close to RM253 (Table 2). The pigmentation in rice requires active regulatory and structural proteins in the anthocyanin biosynthesis pathway. The expression of anthocyanin in rice was reported to be controlled by at least three genes, including the *C-A-P* [35] and the *C-S-A* [1] systems. Within the mapped locus of AZ1711, three candidate genes, *OsC1*, *bHLH*, and *3GT*, were annotated in the anthocyanin biosynthesis pathway [43]. The *OsC1* and *bHLH* genes are regulatory genes because they are TF genes [36]. The MYB (*OsC1*) TF promotes the expression of the *bHLH* gene [44], and the bHLH protein can activate the expression of the *A1* (anthocyanidin synthase) gene to produce pigments. The *3GT* encodes glycosyltransferase, which catalyzes the last step in the anthocyanin pathway [2,45].

The LOC_Os06g10820 locus was annotated as a gene encoding a protein containing the bHLH domain. Although it is a functional gene in the wild-type IR64, its anthocyanin biosynthesis pathway is not expressed because the *OsC1* gene in IR64 is not functional. The pigmentation in AZ1711 is due to the reverse evolution and gain-of-function mutations in the *OsC1* and *3GT* genes. These mutations are unique because the two mutations occurred together in a mapped locus containing three genes in the order, *OsC1*, *bHLH*, and *3GT* [1,35]. Therefore, the *OsC1*-*bHLH*-*3GT* genes in the same locus recover the pigmentation of AZ1711 (Table 4). Intriguingly, the induced mutations in the colorless IR64 made the pigmentation in AZ1711 look like that in the wild rice *O. rufipogon* [46].

A 10 bp insertion was found in the *OsC1* gene of AZ1701, AZ1702, AZ1711, AZ1714, and AZ1715 mutants and resulted in pigmented tissues. The 10 bp fragment is only found in the Nipponbare *japonica* variety but is not presented in the 93-11 and many *indica* varieties. It had never been reported that many *japonica* rice varieties carried the 10 bp insertion while many *indica* rice lost it (Table S4) [36,47]. Some varieties that carried the 10 bp insertion expressed the pigment in tissue, such as *O. rufipogon*, T65 (*japonica*), and ZhanShen97 (*indica*), while Nipponbare (*japonica*) is colorless [36]. The colorlessness in Nipponbare might be due to a 3 bp deletion in the first exon of *OsC1*, resulting in the deletion of amino acid, while no similar deletion was found in 93-11 or IR64 (Figure S3). Furthermore, Nipponbare has a 72 bp deletion that causes loss of function in the *3GT* gene, which is essential for pigmentation (Figure S4). The 10 bp insertion/deletion (In/Del) seems to be a common variation in rice, and it can differentiate most *indica* and *japonica* rice (Table S4) [36], supporting that the variation of 10 bp might occur during evolution before the separation of *indica* and *japonica* rice. The 10 bp in the R3 domain of the MYB DNA binding domain affects the ability of transcription factors to recognize and bind to the structural gene by the frameshift mutation [7,48]. Similarly, the 3 bp deletion caused the amino acid deletion [49] in the R3 DNA binding domain of the MYB. It should affect the recognition of transcription factors and the function of structural genes.

In summary, the 10 bp insertion in the *OsC1* and the 72 bp insertion are found in the *3GT* in the AZ1711, AZ1714, and AZ1715 mutant lines. However, the 72 bp insertion is not found in the pigmented mutant lines AZ1701 and AZ1702, indicating that the 3*GT* gene may not be required for the pigmentation of these lines [6]. The *3GT* gene is near the *OsC1* gene and mutates in many pigmented lines, showing the exact mechanisms by which sodium azide-induced mutations might occur repeatedly and not randomly. According to the bioinformatics analyses, the 72 bp insertion is also found in *O. rufipogon* and 93-11 but not in Nipponbare. The BLASTN analysis found that the 72 bp insertion also presents in the *ORUFI06G07520* gene, which shares 97.2% similarity with the UDP-glycosyltransferase (A0A0E0PV26). Therefore, we speculate that the *3GT* gene might have drifted or been lost during rice domestication or selective breeding for colorless plants, but the structure and function are resumed. The insertional mutations in the NaN_3_-mutagenized mutants recover the function of the proteins, and reverse evolution processes may not occur randomly. A mechanism related to the involvement of retrotransposons might occur and will be discussed later.

### 4.2. The Characteristics of Mutations Induced by Sodium Azide in AZ1711

The pigmented AZ1711 mutant line was generated by sodium azide mutagenesis from the non-pigmented IR64 variety, and insertion was found to be responsible for the color reversal. At the same time, many pigmented mutant lines with the same insertion were found in AZ1701, AZ1702, AZ1714, and AZ1715. Thus, NaN_3_ induced repeated mutations in the mutant pool, as in our previous report that more than 15 blast disease-resistant mutant lines were generated from the TNG67 variety by NaN_3_ mutagenesis [50]. NaN_3_ was reported to induce only point mutations in plants in the past [51,52,53]. In the *OsC1* gene, five-point mutations and a 10 bp insertion, and in the *3GT* gene, 24 point-mutations and a 72 bp insertion, were identified in the pigmented mutant AZ1711 (Figure 3), indicating that not only point mutations but also In/Del mutations can be induced by NaN_3_ in rice.

Interestingly, the insertions in two pigmentation genes from a single locus of the AZ1711 show the pigment trait similar to that in the wild rice *O. rufipogon*. In addition, the four mutants, AZ1701, AZ1702, AZ1714, and AZ1715 also have the same mutations and recover functions to produce pigment. Therefore, mutations induced by sodium azide are not random but show a trend of reverse evolution; they can be considered reverse evolution events because the colorless trait of plants has been selected during domestication or selective breeding. Our results showed that the 10 bp insertion fragment in Chr. 6, identified in AZ1711, is presented in the *japonica* variety Nipponbare and the W1943 line of wild rice (*O. rufipogon*), but is not found in *indica* varieties IR64 and 93-11 (Figure 3A). Similarly, the 72 bp insertional fragment is discovered in the *3GT* gene in AZ1711, *indica* rice 93-11 (Figure 3B), and the W1943 strain but has no function in IR64 and Nipponbare without color on plants.

Since Asia rice, like japonica and indica types, come from the exact origin, their traits and genomes might evolve with either the gain or loss function during their evolution. In this study, we discovered two fragments in the mutant AZ1711 that do not exist in its parental variety, IR64. However, the same or similar fragments can be identified on other chromosomes of IR64 (Figure 5 and Figure 6). The *indica* rice variety IR64 lost some traits like seed dormancy, awn, pigment, and so on that had evolved from its ancient rice origin [46,54,55]. The recovery of gene functions induced by gene insertion in our anthocyanin mutants is assumed reverse evolution mutation from the wild-type IR64 through NaN_3_ treatment.

The reverse evolution might not occur randomly because of the 10 bp insertion found in the *japonica* variety Nipponbare and the 72 bp fragment found in the *indica* variety 93-11 in the genome database of these two subtype rice varieties and, instead, might have occurred as repetitive events in the mutation pool. Unlike the DNA repair by CRISPR that is generated randomly [56], our results indicated that the probability of this mutation is too high to be explained by random chance. The large fragment mutation does not quickly happen in the induced mutations, while most reported cases were by the insertion of TEs or T-DNA mechanisms [57,58]. The 72 bp insertion and its sequences also match (homologous) to the part of retrotransposons that might reflect possible mechanisms (Figure 6). Transposable elements (TEs) play essential roles in plant evolution with un-identified functions, and no report has ever related it to the mutations induced by sodium azide. Therefore, direct evidence is required to prove the involvement of the TEs in sodium azide-induced mutations.

### 4.3. Proposed Mechanisms for Sodium Azide Mutagenesis

NaN_3_ was reported to provide the oxidative stress condition in mutation treatment to the plant tissues [59,60,61]. It has been reported that it introduces point mutation by adding methyl or ethyl groups to bases, resulting in base mispairing during DNA replication [62]. Our results showed that NaN_3_ induced not only point mutations but also insertions.

According to the sequencing results of the candidate genes, insertion seems to happen frequently. The sequence of the 72 bp insertional fragment is 80–90% similar to the sequences of one-gene family *Ty1*-*Copia* retrotransposon (LOC_Os04g12090) on Chr. 4 with a high identity (83–90%) (Figure 6). In addition, a secondary structure was found when analyzing the 72 bp insertion (Figure 7). The very unusual secondary structures of the 72 bp insertional fragment were analyzed as part of *Ty1-Copia* retrotransposon sequences in the mutant line AZ1711, providing evidence that retrotransposons might have been activated during mutagenesis and integrated or recombined into the original rice genome during DNA replication. Our result may be the first finding of retrotransposon genomic sequences inserted in the genes induced by sodium azide treatment in rice. Unraveling the mechanism and its relationship with mutations induced by sodium azide will be interesting. Unfortunately, no direct evidence can prove the hypothesis or provide direct evidence to show that the TEs can be activated to induce mutations by external treatments of sodium azide so far. In regard to the previous report, it was the first time we realized that *Tos17* is active in the rice genome and affects the phenotype after tissue culture [18]. Our finding showed progress in understanding that the retrotransposon also can be activated by NaN_3_ mutagenesis. Moreover, this activation is related to the genes, which link to rice evolution. Transposable elements and the phenotypes they introduced in the mutant kernels were first observed by Dr. Barbara McClintock in maize in “The origin and behavior of mutable loci in maize” in the early 90s [63]. The association of TE insertion and rice grain width was found in the 1132 accessions rice [64]. The insertional fragments from retrotransposon might be a mechanism of functional recovery mutation as reverse mutation [65]. It had been reported that the *Copia*-*like* retrotransposon *Tos17* in rice was activated by tissue culture and generated 47,196 *Tos17*-insertional rice mutants. In addition, the *Tos17* insertion in genic regions was three-fold higher than in intergenic regions [66]. The retrotransposons may be activated by the oxidative stress-induced double-strand breakages (DSBs) [67] during the sodium azide mutagenesis conditions at low pH. The homologous recombination repair mechanism in the genome occurred and produced the insertion of sequences with high nucleotide identity, or the transposon was activated and intruded into the gene through the unusual secondary structure of DNA sequences [68]. Due to the stress, the ends-out recombination was reported to occur between two paired homologous sequences located on different chromosomes [69,70]. It is reasonable to speculate that sodium azide induces oxidative stress during seed treatment at a low pH and activates retrotransposons or other TEs, and causes many mutations and diverse phenotypes in the rice genome. The TNG67 variety has only one copy of *Copia*-LTR (B4-450); however, its mutants show seven restriction fragment length polymorphisms (RFLPs), and many mutants (unpublished data) have more than one copy, as discovered with the *Tos17* copy number that increased in rice tissue culture [18]. Due to the stress, the ends-out recombination was reported to occur between two paired homologous sequences located on different chromosomes [71].

According to the high sequences identity around the insertional fragment, it is reasonable to explain that this insertion may be inserted into the *3GT* through the homologous recombination during DNA replication or repair after sodium azide mutagenesis through the error-prone non-homologous end-joining (NHEJ) mechanism. It has been reported that the NHEJ could be more precise in repairing the DBS and may result in a diversity of sequence outcomes [72]. However, the insertional sequences in the AZ1711 are the same as the homolog one in the genome of the retrotransposon containing the 72 bp *3GT* sequences (>83% identity). In a previous report, at least 74% identity is enough to cross over in yeast [73]. Therefore, the insertional fragment might have been inserted through the activation of RT through the single-strand annealing (SSA) mechanism to introduce the insertional sequence [72] and repaired by end-joining as a regular one.

Due to the stress, the ends-out recombination was reported to occur between two paired homologous sequences located on different chromosomes [74]. However, the detailed mechanism for retrotransposon-induced mutation must be further evidenced.

## 5. Conclusions

In the sodium azide-induced mutant pool of the rice variety IR64, there are mutant lines with many trait variations, among which the colored mutants have apparent color changes. Several pigmented lines were selected for genetics analysis. The particular locus was mapped on Chr. 6 and carried three candidate genes, *MYB* (*OsC1*), *bHLH*, and *3GT,* within ~0.83 Mb inherited as one locus. The variations found in the regulatory gene *OsC1* and the structural gene *3GT,* which belonged to the anthocyanin biosynthesis pathway, differed between AZ1711 and its wild-type IR64, and their sequence differences were confirmed to be the dominant characteristics. The insertions of 10 bp and 72 bp in the *OsC1* and *3GT* genes to restore the protein function in the IR64 background of the mutant lines might not randomly occur. Because two types of rice varieties, Nipponbare and 93-11, carry a similar insertion, these results support the reverse evolution mutations induced by sodium azide mutagenesis. The sequence and structure analysis of insertion fragments demonstrate that the reverse evolution might relate to the action of retrotransposon during the mutagenesis of sodium azide. The similar mutations on many mutant lines and recovery of the protein functions supported our hypothesis that sodium azide-induced mutations may not randomly happen. The anthocyanin is an antioxidant because of its ring structure. Therefore, the pigmented rice must have a higher nutritional quality than non-pigment rice. This shows an impact on generating new functional rice varieties using NaN_3_ mutagenesis. Finally, the SSR markers during gene identification can be used for breeding programs.

## Figures and Tables

**Table 1:** Pigmentations in four tissues of IR64 and its pure line mutants and their F_1_ and F_2_ plants.

Plant		Tissue Phenotype ^a^			
		Leaf-Sheath	Auricle	Ligule	Apiculus
IR64		G	G	G	G
AZ1701		P	LP	P	R
AZ1702		P	LG	P	R
AZ1711		P	LP	P	R
AZ1714		LP	P	P	R
AZ1715		P	P	P	R
AZ1701×IR64 F_1_		P	LP	LP	R
AZ1702×IR64 F_1_		P	LP	LP	R
AZ1711×IR64 F_1_		P	P	P	P
AZ1714×IR64 F_1_		P	P	P	R
AZ1715×IR64 F_1_		P	P	P	R
AZ1711×IR64 F_2_	Pig: Non-pig ^b^	402:155	401:156	400:157	401:156
	Tested ratio	3:1	3:1	3:1	3:1
	*χ* ^2^	2.45 ^ns^	2.77 ^ns^	3.10 ^ns^	2.77 ^ns^

^a^ Color: G, green; P, purple; LP, light purple; R, red. ^b^ Pig, pigmentation; non-pig, non-pigmentation. ^ns^ Means that the observed phenotypic ratio matches the expected genetic ratio. *χ*^2^_(0.05, 1)_ = 3.841.

**Table 2:** ANOVA analysis, *R*^2^, additive, and the dominant effect of pigmentation trait with markers in the RM276-RM6817 region were analyzed using the MapDisto with the AZ1711×IR64 F_2_ mapping population.

Marker	*p*-Value ^a^	*R* ^2 b^	A ^c^	D ^d^	|D/A| ^e^
RM6773	1.41 × 10^−11^	0.43	−0.33	0.34	1.02
RM5754	2.11 × 10^−11^	0.42	−0.36	0.3	0.83
RM253	7.24 × 10^−31^	0.78	−0.45	0.46	1.03
RM2615	2.78 × 10^−18^	0.59	−0.40	0.37	0.94

^a^ Probability value of *t*-test. ^b^ Coefficient of determination. ^c^ Additive effect. ^d^ dominant effect. ^e^ Gene action is determined on the basis of genotypic class means |D/A|; where <0.5 = additive, >0.5–<0.75 = partial dominant, >0.75–<1.25 = dominant, and >1.25 = over-dominant.

**Table 3:** The candidate genes identified in the mapped region involved in the pigmentation of the AZ1711 mutant line of IR64 cultivar.

Gene	MSU ID	BGI ID	Annotation
*OsC1*	LOC_Os06g10350	BGIOSGA022508	MYB family transcription factor
*bHLH*	LOC_Os06g10820	BGIOSGA022533	helix-loop-helix DNA-binding domain-containing protein
*3GT*	LOC_Os06g11270	BGIOSGA021671	anthocyanidin 3-O-glucosyltransferase

**Table 4:** Mapping the mutated pigmentation loci for rice tissues using SSR markers of the IR64 variety, F_1_ plant, F_2_ population of AZ1711, and IR64 through R/qtl.

Plant	RM5754	C	OsC1	RM253	S	G	3Oglu	RM2615	Phenotype
AZ1711	AA	CC	AA	AA	SS	GG	AA	AA	Purple
AZ1711×IR64 F_1_ ^a^	AB	Cc	AB	AB	SS	Gg	AB	AB	Purple
F_2_ P13	AA	CC	AA	AA	SS	GG	AA	AA	Purple
F_2_ G11	AB	cc	BB	BB	SS	gg	BB	BB	Green
F_2_ G15	BB	cc	BB	BB	SS	Gg	AB	AB	Green
IR64	BB	cc	BB	BB	SS	gg	BB	BB	Green

^a^ F_1_, P13, G11, and G15 are F_2_ plants of the AZ1711×IR64 cross randomly chosen for genotype testing within two mapped markers, RM5754 and RM2615. Only genotypes showing dominant C (OsC1), S (bHLH), and G (3GT) presented purple; otherwise, they were green.

## Data Availability

Data is contained within the article or Supplementary Material.

## Supplementary Materials

The following supporting information can be downloaded at: https://www.mdpi.com/article/10.3390/cimb46120795/s1, Figure S1: Plant architecture of AZ1701, AZ1702, AZ1714, and AZ1715 mutants at the mature stage and its grain; Figure S2: Linkage map of the markers applied on 92 F2 individuals of the AZ1711×IR64 cross; Figure S3: Nucleotide and amino acid sequence of OsC1 gene; Figure S4: Nucleotide and amino acid sequence of 3GT gene; Table S1: The SSR markers were applied in polymorphism screening; Table S2: The annotated and candidate genes from japonica and indica database; Table S3: Sequence of primers used in this study; Table S4: The appearances of 10 bp insertion and pigmentation in *japonica* and *indica* rice varieties.

## References

[1] Sun X.M., Zhang Z.Y., Chen C., Wu W., Ren N.N., Jiang C.H., Yu J.P., Zhao Y., Zheng X.M., Yang Q.W. (2018). The C–S–A gene system regulates hull pigmentation and reveals evolution of anthocyanin biosynthesis pathway in rice. J. Exp. Bot..

[2] Xia D., Zhou H., Wang Y.P., Li P.B., Fu P., Wu B., He Y.Q. (2021). How rice organs are colored: The genetic basis of anthocyanin biosynthesis in rice. Crop J..

[3] Liu Y., Tikunov Y., Schouten R.E., Marcelis L.F.M., Visser R.G.F., Bovy A. (2018). Anthocyanin biosynthesis and degradation mechanisms in Solanaceous vegetables: A review. Front. Chem..

[4] Zha J., Koffas M. (2017). Production of anthocyanins in metabolically engineered microorganisms: Current status and perspectives. Synth. Syst. Biotechnol..

[5] Outchkourov N.S., Karlova R., Hölscher M., Schrama X., Blilou I., Jongedijk E., Simon C.D., van Dijk A.D.J., Bosch D., Hall R.D. (2018). Transcription factor-mediated control of anthocyanin biosynthesis in vegetative tissues. Plant Physiol..

[6] Zhu Q.L., Yu S.Z., Zeng D.C., Liu H.M., Wang H.C., Yang Z.F., Xie X.R., Shen R.X., Tan J.T., Li H.Y. (2017). Development of “purple endosperm rice” by engineering anthocyanin biosynthesis in the endosperm with a high-efficiency transgene stacking system. Mol. Plant.

[7] Meng L.Z., Qi C.Y., Wang C.H., Wang S., Zhou C.L., Ren Y.L., Cheng Z.J., Zhang X., Guo X.P., Zhao Z.C. (2021). Determinant factors and regulatory systems for anthocyanin biosynthesis in rice apiculi and stigmas. Rice.

[8] Khan R.A., Abbas N. (2023). Role of epigenetic and post-translational modifications in anthocyanin biosynthesis: A review. Gene.

[9] Gruszka D., Szarejko I., Maluszynski M. (2012). Sodium azide as a mutagen. Plant Mutation Breeding and Biotechnology.

[10] Udage A. (2021). Introduction to plant mutation breeding: Different approaches and mutagenic agents. J. Agric. Sci.-Sri Lanka.

[11] Wyss O., Clark J.B., Haas F., Stone Wilson S. (1948). The role of peroxide in the biological effects of irradiated broth. J. Bacteriol..

[12] Ilhan E., Kasapoğlu A., Türkoğlu A., Aygören A., Muslu S., Aydin M., Aldaif M., Haliloglu K. (2023). Evaluation of sodium azide as a chemical mutagen in developing cold-tolerant quinoa (*Chenopodium quinoa* Willd.) lines. Iran. J. Sci. Technology. Trans. A Sci..

[13] Maan S., Brar J. (2021). Mutagenic sensitivity analysis in guava (*Psidium guajava* L.). Fruits.

[14] Punia S., Singh M., Grover G., Tiwari C. (2022). Tilling: A reverse genetics technique. Current Innovations in Genetics and Plant Breeding.

[15] Srivastava P., Marker S., Pandey P., Tiwari D.K. (2011). Mutagenic effects of sodium azide on the growth and yield characteristics in wheat (*Triticum aestivum* L. em. Thell.). Asian J. Plant Sci..

[16] Horváth V., Merenciano M., González J. (2017). Revisiting the relationship between transposable elements and the Eukaryotic stress response. TiG.

[17] Liu Z., Zhao H., Yan Y., Wei M.X., Zheng Y.C., Yue E., Alam M., Smartt K., Duan M.H., Xu J.H. (2021). Extensively current activity of transposable elements in natural rice accessions revealed by singleton insertions. Front. Plant Sci..

[18] Hirochika H., Sugimoto K., Otsuki Y., Tsugawa H., Kanda M. (1996). Retrotransposons of rice involved in mutations induced by tissue culture. Proc. Natl. Acad. Sci. USA.

[19] Rasik S., Raina A., Laskar R.A., Wani M.R., Reshi Z.A., Khan S., Ndhlala A.R. (2022). Lower doses of sodium azide and methyl methanesulphonate improved yield and pigment contents in vegetable cowpea [*Vigna unguiculata* (L.) Walp.]. S. Afr. J. Bot..

[20] Türkoğlu A., Haliloğlu K., Tosun M., Szulc P., Demirel F., Eren B., Bujak H., Karagöz H., Selwet M., Özkan G. (2023). Sodium azide as a chemical mutagen in wheat (*Triticum aestivum* L.): Patterns of the genetic and epigenetic effects with iPBS and CRED-iPBS techniques. Agriculture.

[21] Kobayashi S., Goto-Yamamoto N., Hirochika H. (2004). Retrotransposon-induced mutations in grape skin color. Science.

[22] Butelli E., Licciardello C., Zhang Y., Liu J.J., Mackay S., Bailey P., Reforgiato-Recupero G., Martin C. (2012). Retrotransposons control fruit-specific, cold-dependent accumulation of anthocyanins in blood oranges. Plant Cell.

[23] Lu T.W., Chen W.H., Chen P.Y., Shu Y.C., Chen H.H. (2024). Perturbation of periodic spot-generation balance leads to diversified pigmentation patterning of harlequin *Phalaenopsis orchids*: In silico prediction. BMC Plant Biol..

[24] Zheng J., Wu H., Zhu H.B., Huang C.Y., Liu C., Chang Y.S., Kong Z.C., Zhou Z.H., Wang G.W., Lin Y.J. (2019). Determining factors, regulation system, and domestication of anthocyanin biosynthesis in rice leaves. New Phytol..

[25] Wang C.S., Tseng T.H., Lin C.Y. (2002). Rice biotech research at the taiwan agricultural research institute. Asia-Pac. Biotech News.

[26] Jeng T.L., Lin Y.W., Wang C.S., Sung J.M. (2012). Comparisons and selection of rice mutants with high iron and zinc contents in their polished grains that were mutated from the indica type cultivar IR64. J. Food Compos. Anal..

[27] IRRI (2013). Standard Evaluation System for Rice.

[28] Doyle J.J., Dickson E.E. (1987). Preservation of plant samples for DNA restriction endonuclease analysis. Taxon.

[29] Tseng H.Y., Lin D.G., Hsieh H.Y., Tseng Y.J., Tseng W.B., Chen C.W., Wang C.S. (2015). Genetic analysis and molecular mapping of QTLs associated with resistance to bacterial blight in a rice mutant, SA0423. Euphytica.

[30] Temnykh S., DeClerck G., Lukashova A., Lipovich L., Cartinhour S., McCouch S. (2001). Computational and experimental analysis of microsatellites in rice (*Oryza sativa* L.): Frequency, length variation, transposon associations, and genetic marker potential. Genome Res..

[31] McCouch S.R., Teytelman L., Xu Y.B., Lobos K.B., Clare K., Walton M., Fu B.Y., Maghirang R., Li Z.K., Xing Y.Z. (2002). Development and mapping of 2240 new SSR markers for rice (*Oryza sativa* L.). DNA Res..

[32] Broman K.W., Wu H., Sen Ś., Churchill G.A. (2003). R/qtl: QTL mapping in experimental crosses. Bioinformatics.

[33] Akond Z., Alam M.J., Hasan M.N., Uddin M.S., Alam M., Mollah M.N.H. (2019). A comparison on some interval mapping approaches for QTL detection. Bioinformation.

[34] Lorieux M. (2012). MapDisto: Fast and efficient computation of genetic linkage maps. Mol. Breed..

[35] Takahashi M. (1957). Analysis on apiculus color genes essential to anthocyanin coloration rice. J. Fac. Agric. Hokkaido Univ..

[36] Saitoh K., Onishi K., Mikami I., Thidar K., Sano Y. (2004). Allelic diversification at the C (OsC1) locus of wild and cultivated rice: Nucleotide changes associated with phenotypes. Genetics.

[37] Sweeney M.T., Thomson M.J., Pfeil B.E., McCouch S. (2006). Caught red-handed: Rc encodes a basic helix-loop-helix protein conditioning red pericarp in rice. Plant Cell.

[38] Yang X.H., Xia X.Z., Zhang Z.Q., Nong B.X., Zeng Y., Wu Y.Y., Xiong F.Q., Zhang Y.X., Liang H.F., Pan Y.H. (2019). Identification of anthocyanin biosynthesis genes in rice pericarp using PCAMP. Plant Biotechnol. J..

[39] Mohan M., Nair S., Bhagwat A., Krishna T.G., Yano M., Bhatia C.R., Sasaki T. (1997). Genome mapping, molecular markers and marker-assisted selection in crop plants. Mol. Breed..

[40] Young N.D., Phillips R.L., Vasil I.K. (1994). Constructing a plant genetic linkage map with DNA markers. DNA-Based Markers in Plants.

[41] Yue B., Cui K.H., Yu S.B., Xue W.Y., Luo L.J., Xing Y.Z. (2006). Molecular marker-assisted dissection of quantitative trait loci for seven morphological traits in rice (*Oryza sativa* L.). Euphytica.

[42] Olsen O., Wang X., von Wettstein D. (1993). Sodium azide mutagenesis: Preferential generation of AT-->GC transitions in the barley Ant18 gene. Proc. Natl. Acad. Sci. USA.

[43] Dooner H.K., Robbins T.P., Jorgensen R.A. (1991). Genetic and developmental control of anthocyanin biosynthesis. Annu. Rev. Genet..

[44] Koes R., Verweij W., Quattrocchio F. (2005). Flavonoids: A colorful model for the regulation and evolution of biochemical pathways. Trends Plant Sci..

[45] Grotewold E. (2006). The genetics and biochemistry of floral pigments. Annu. Rev. Plant Biol..

[46] Ishikawa R., Castillo C.C., Fuller D.Q. (2020). Genetic evaluation of domestication-related traits in rice: Implications for the archaeobotany of rice origins. Archaeol. Anthropol. Sci..

[47] Choudhury B.I., Khan M.L., Dayanandan S. (2014). Patterns of nucleotide diversity and phenotypes of two domestication related genes (OsC1 and Wx) in indigenous rice varieties in Northeast India. BMC Genet..

[48] Qiao W.H., Wang Y.Y., Xu R., Yang Z.Y., Sun Y., Su L., Zhang L.Z., Wang J.R., Huang J.F., Zheng X.M. (2021). A functional chromogen gene C from wild rice is involved in a different anthocyanin biosynthesis pathway in indica and japonica. Theor. Appl. Genet..

[49] Shatunov A., Olivé M., Odgerel Z., Stadelmann-Nessler C., Irlbacher K., van Landeghem F., Bayarsaikhan M., Lee H.S., Goudeau B., Chinnery P.F. (2009). In-frame deletion in the seventh immunoglobulin-like repeat of filamin C in a family with myofibrillar myopathy. Eur. J. Hum. Genet..

[50] Wang C.S., Lo K.L., Wang A.Z. (2019). Sodium azide mutagenesis generated diverse and broad spectrum blast resistance mutants in rice. Euphytica.

[51] Kleinhofs A., Smith J.A. (1976). Effect of excision repair on azide-induced mutagenesis. Mutat. Res..

[52] Thomas H.T., Areum C., Isabelle M.H., Kathie J.N., Diana B.-W. (2016). Effectiveness of sodium azide alone compared to sodium azide in combination with methyl nitrosurea for rice mutagenesis. Plant Breed. Biotechnol..

[53] Viana V.E., Pegoraro C., Busanello C., Costa de Oliveira A. (2019). Mutagenesis in rice: The basis for breeding a new super plant. Front. Plant Sci..

[54] Lu Y.Q., Xu Y.Z., Li N. (2022). Early domestication history of Asian rice revealed by mutations and genome-wide analysis of gene genealogies. Rice.

[55] Stein J.C., Yu Y.S., Copetti D., Zwickl D.J., Zhang L., Zhang C.J., Chougule K., Gao D.Y., Iwata A., Goicoechea J.L. (2018). Genomes of 13 domesticated and wild rice relatives highlight genetic conservation, turnover and innovation across the genus Oryza. Nat. Genet..

[56] Zhu Y.W., Lin Y.R., Chen S.B., Liu H.Q., Chen Z.J., Fan M.Y., Hu T.J., Mei F.T., Chen J.M., Chen L. (2019). CRISPR/Cas9-mediated functional recovery of the recessive rc allele to develop red rice. Plant Biotechnol. J..

[57] Banakar R., Eggenberger A.L., Lee K., Wright D.A., Murugan K., Zarecor S., Lawrence-Dill C.J., Sashital D.G., Wang K. (2019). High-frequency random DNA insertions upon co-delivery of CRISPR-Cas9 ribonucleoprotein and selectable marker plasmid in rice. Sci. Rep..

[58] Gong W.K., Zhou Y., Wang R., Wei X.L., Zhang L., Dai Y., Zhu Z. (2021). Analysis of T-DNA integration events in transgenic rice. J. Plant Physiol..

[59] Garg M., Poornima G., Rajyaguru P.I. (2020). Elucidation of the RNA-granule inducing sodium azide stress response through transcriptome analysis. Genomics.

[60] Wilson D.F., Chance B. (1967). Azide inhibition of mitochondrial electron transport I. The aerobic steady state of succinate oxidation. Biochim. Biophys. Acta Bioenerg..

[61] Carpentier M.-C., Manfroi E., Wei F.J., Wu H.P., Lasserre E., Llauro C., Debladis E., Akakpo R., Hsing Y.I., Panaud O. (2019). Retrotranspositional landscape of Asian rice revealed by 3000 genomes. Nat. Commun..

[62] Oladosu Y., Rafii M.Y., Abdullah N., Hussin G., Ramli A., Rahim H.A., Miah G., Usman M. (2016). Principle and application of plant mutagenesis in crop improvement: A review. Biotechnol. Biotechnol. Equip..

[63] McClintock B. (1950). The origin and behavior of mutable loci in maize. Proc. Natl. Acad. Sci. USA.

[64] Roland A., Marie-Christine C., Hsing Y.I., Olivier P. (2020). The impact of transposable elements on the structure, evolution and function of the rice genome. New Phytol..

[65] Ishikawa S., Ishimaru Y., Igura M., Kuramata M., Abe T., Senoura T., Hase Y., Arao T., Nishizawa N.K., Nakanishi H. (2012). Ion-beam irradiation, gene identification, and marker-assisted breeding in the development of low-cadmium rice. Proc. Natl. Acad. Sci. USA.

[66] Miyao A., Tanaka K., Murata K., Sawaki H., Takeda S., Abe K., Shinozuka Y., Onosato K., Hirochika H. (2003). Target site specificity of the Tos17 retrotransposon shows a preference for insertion within genes and against insertion in retrotransposon-rich regions of the genome. Plant Cell.

[67] Farkash E.A., Prak E.T.L. (2006). DNA damage and L1 retrotransposition. J. Biomed. Biotechnol..

[68] Kikuchi K., Terauchi K., Wada M., Hirano H.Y. (2003). The plant MITE mPing is mobilized in anther culture. Nature.

[69] Li X., Heyer W.-D. (2008). Homologous recombination in DNA repair and DNA damage tolerance. Cell Res..

[70] Ray A., Langer M. (2002). Homologous recombination: Ends as the means. Trends Plant Sci..

[71] Colot V., Haedens V., Rossignol J.-L. (1998). Extensive, nonrandom diversity of excision footprints generated by Ds-Like transposon Ascot-1 suggests new parallels with V(D)J recombination. Mol. Cell. Biol..

[72] Pannunzio N.R., Watanabe G., Lieber M.R. (2018). Nonhomologous DNA end-joining for repair of DNA double-strand breaks. J. Biol. Chem..

[73] Datta A., Hendrix M., Lipsitch M., Jinks-Robertson S. (1997). Dual roles for DNA sequence identity and the mismatch repair system in the regulation of mitotic crossing-over in yeast. Proc. Natl. Acad. Sci. USA.

[74] Sukegawa S., Toki S., Saika H. (2022). Genome editing technology and its application to metabolic engineering in rice. Rice.

