# Supplementary material for: Reverse Mutations in Pigmentation Induced by Sodium Azide in the IR64 Rice Variety
Source: Curr Issues Mol Biol. 2024 Nov 22;46(12):13328–46. doi: 10.3390/cimb46120795 (PMC11727009; doi:10.3390/cimb46120795)
Supplement: Supplementary file 1 [file cimb-46-00795-s001.zip › 20241010 Sup Figure for cimb KJ.pdf]

Supporting Information

Article title: Reverse pigmentation mutations induced by sodium azide in the IR64 rice variety

Authors: Hsian-Jun Chen, Anuchart Sawasdee, Yu-Ling Lin, Min-Yu Chiang, Hsin-Yi Chang, Wen-Hsiung Li, Chang-Sheng Wang

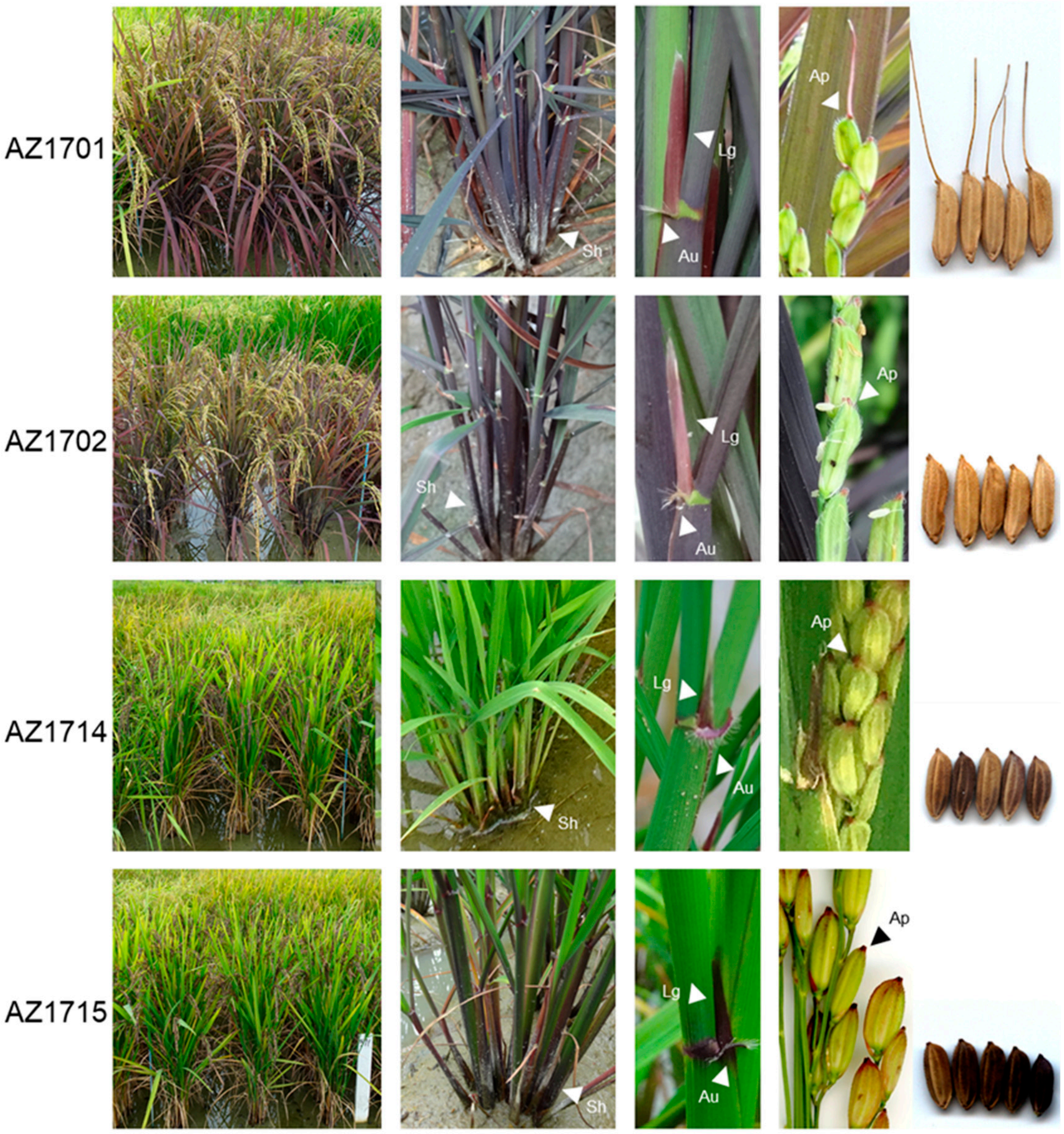

Figure S1. Plant architecture of AZ1701, AZ1702, AZ1714, and AZ1715 mutants at the mature stage and its grain. Auricle (Au), ligule (Lg), apiculus (Ap), and sheath (Sh).

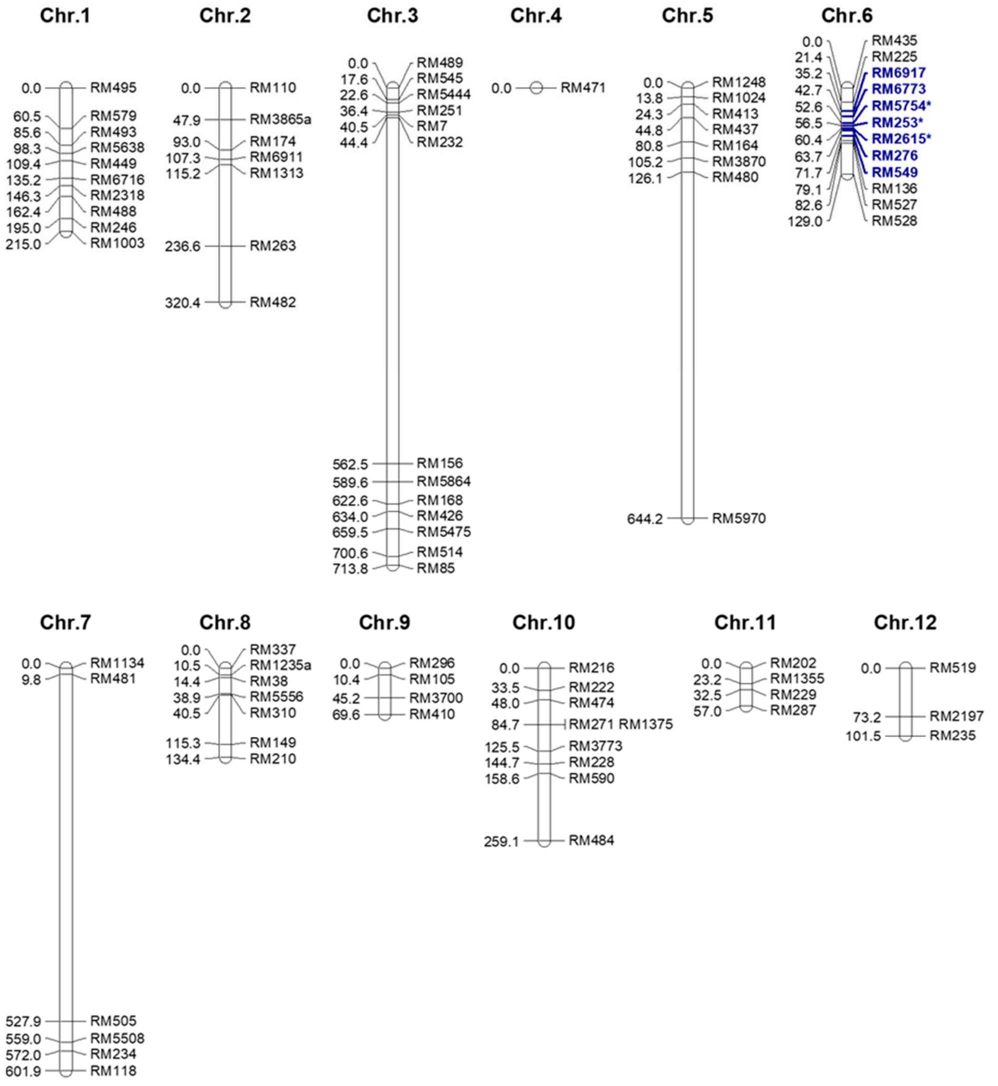

Figure S2. Linkage map of the markers applied on 92 F_2 individuals of the AZ1711×IR64 cross. The seven additional markers for fine mapping were labeled in blue. The locus responsible for the pigmentation was fine mapped among the starred markers.

```
                                          |----------------------------
Nipponbare ATGGGGAGGAGAGCTTGCTGCGCAAAGGAAGGGATGAAGAGAGGGGCATGGACGAGCAAG (60)
9311       ATGGGGAGGAGAGCTTGCTGCGCAAAGGAAGGGATGAAGAGAGGGGCATGGACGAGCAAG
IR64       ATGGGGAGGAGAGCTTGCTGCGCAAAGGAAGGGATGAAGAGAGGGGCATGGACGAGCAAG
AZ1711     ATGGGGAGGAGAGCTTGCTGCGCAAAGGAAGGGATGAAGAGAGGGGCATGGACGAGCAAG
           ************************************************************
           M  G  R  R  A  C  C  A  K  E  G  M  K  R  G  A  W  T  S  K   (20)

           ------------------------ R2 residues -----------------------
Nipponbare GAGGACGACGTGCTTGCCTCCTACATCAAGTCCCATGGCGAAGGCAAGTGGCGCGAGGTC (120)
9311       GAGGACGACGTGCTTGCCTCCTACATCAAGTCCCATGGCGAAGGCAAGTGGCGCGAGGTC
IR64       GAGGACGACGTGCTTGCCTCCTACATCAAGTCCCATGGCGAAGGCAAGTGGCGCGAGGTC
AZ1711     GAGGACGACGTGCTTGCCTCCTACATCAAGTCCCATGGCGAAGGCAAGTGGCGCGAGGTC
           ************************************************************
           E  D  D  V  L  A  S  Y  I  K  S  H  G  E  G  K  W  R  E  V   (40)

           ---------------
Nipponbare CCCCAACGAGCTGGTGAGCTAGCTATTACCTAATCGATCGATGGTCATCGATCATGAGAT (180)
9311       CCCCAACGAGCTGGTGAGCTAGCTATTACCTAATCGATCGATGGTCATCGATCATGAGAT
IR64       CCCCAACGAGCTGGTGAGCTAGCTATTACCTAATCGATCGATGGTCATCGATCATGAGAT
AZ1711     CCCCAACGAGCTGGTGAGCTAGCTATTACCTAATCGATCGATGGTCATCGATCATGAGAT
           ************************************************************
           P  Q  R  A  G                                                (45)

Nipponbare GATGATGATGAGATTTGTACTTAATTGTGATCTGTATGGATGCTGTTGTTGATCAAGTTC (240)
9311       GATGATGATGAGATTTGTACTTAATTGTGATCTGTATGGATGCTGTTGTTGATCAAGTTC
IR64       GATGATGATGAGATTTGTACTTAATTGTGATCTGTATGGATGCTGTTGTTGATCAAGTTC
AZ1711     GATGATGATGAGATTTGTACTTAATTGTGATCTGTATGGATGCTGTTGTTGATCAAGTTC
           ************************************************************

                                             ---------- R2 residues -----
Nipponbare TTGCGATCGATCGATCTGAATTTTCAGGTTTGAGGCGGTGCGGCAAGAGCTGCAGGCTCC (300)
9311       TTGCGATCGATCGATCTGAATTTTCAGGTTTGAGGCGGTGCGGCAAGAGCTGCAGGCTCC
IR64       TTGCGATCGATCGATCTGAATTTTCAGGTTTGAGGCGGTGCGGCAAGAGCTGCAGGCTCC
AZ1711     TTGCGATCGATCGATCTGAATTTTCAGGTTTGAGGCGGTGCGGCAAGAGCTGCAGGCTCC
           ************************************************************
                                             L  R  R  C  G  K  S  C  R  L  R (56)

           -------------------------|----------------------------------
Nipponbare GGTGGCTCAACTATCTCCGGCCTAACATCAAGCGCGGCAACATCGACGACGACGAGGAG- (359)
9311       GGTGGCTCAACTATCTCCGGCCTAACATCAAGCGCGGCAACATCGACGACGACGAGGAGG (360)
IR64       GGTGGCTCAACTATCTCCGGCCTAACATCAAGCGCGGCAACATCGACGACGACGAGGAGG (360)
AZ1711     GGTGGCTCAACTATCTCCGGCCTAACATCAAGCGCGGCAACATCGACGACGACGAGGAGG (360)
           ***********************************************************
Nipponbare   W  L  N  Y  L  R  P  N  I  K  R  G  N  I  D  D  D  E  E  -  (75)
9311         W  L  N  Y  L  R  P  N  I  K  R  G  N  I  D  D  D  E  E  E  (76)
IR64         W  L  N  Y  L  R  P  N  I  K  R  G  N  I  D  D  D  E  E  E  (76)
AZ1711       W  L  N  Y  L  R  P  N  I  K  R  G  N  I  D  D  D  E  E  E  (76)
```

```
            ───────────── R3 residues ─────────────
Nipponbare --CTCATCGTCAGGCTCCACACCCTCCTCGGCAACAGGTAATCTCATCACTTCATGATCA (417)
9311       AGCTCATCGTCAGGCTCCACACCCTCCTCGGCAACAGGTAATCTCATCACTTCATGATCA (420)
IR64       AGCTCATCGTCAGGCTCCACACCCTCCTCGGCAACAGGTAATCTCATCACTTCATGATCA (420)
AZ1711     AGCTCATCGTCAGGCTCCACACCCTCCTCGGCAACAGGTAATCTCATCACTTCATGATCA (420)
           ************************************************************
Nipponbare   L  I  V  R  L  H  T  L  L  G  N  R  W                      (88)
9311         L  I  V  R  L  H  T  L  L  G  N  R  W                      (89)
IR64         L  I  V  R  L  H  T  L  L  G  N  R  W                      (89)
AZ1711       L  I  V  R  L  H  T  L  L  G  N  R  W                      (89)

Nipponbare CTCCGAGTTCCGTATCAATTTCGTTGAGTTCACAGCTTAAATTTGGAGCTATTTGGTACT (477)
9311       CTCCGAGTTCCGTATCAATTTCGTTGAGTTCACAGCTTAAATTTGGAGCTATTTGGTACT (480)
IR64       CTCCGAGTTCCGTATCAATTTCGTTGAGTTCACAGCTTAAATTTGGAGCTATTTGGTACT (480)
AZ1711     CTCCGAGCTCCGTATCAATTTCGTTGAGTTCACAGCTTAAATTTGGAGCTATTTGGTACT (480)
           ******* ****************************************************

Nipponbare GTCGGTGTGTGGATAGTGATATACTTTTTTCTTTTCCTGGTTACGGCTTTTTAGGGTTGT (537)
9311       GTCGGTGTGTGGATAGTGATATACTTTTTTCTTTTCCTGGTTACGGCTTTTTAGGGTTGT (540)
IR64       GTCGGTGTGTGGATAGTGATATACTTTTTTCTTTTCCTGGTTACGGCTTTTTAGGGTTGT (540)
AZ1711     GTCGGTGTGTGGATAGTGATATACTTTTTTCTTTTCCTGGTTACGGCTTTTTAGGGTTGT (540)
           ************************************************************

Nipponbare AATATAAACTTCGCAACTTCTTATACGAGTCATTCGAAAAAAATTTGAAGCTGGCTAACG (697)
9311       AATATAAACTTCGCAACTTCTTATACGAGTCATTCGAAAAAAATTTGAAGCTGGCTAACG (600)
IR64       AATATAAACTTCGCAACTTCTTATACGAGTCATTCGAAAAAAATTTGAAGCTGGCTAACG (600)
AZ1711     AATATAAACTTCACAACTTCTTATACGAGTCATTCGAAAAAAATTTGGAGCTGGCTAACG (600)
           ************ ********************************** ************

Nipponbare CTAGACAATAATAAGCTGATTTAACTTCTGTTTTATTTTTATTTATTTTTATCTTTATCT (657)
9311       CTAGACAATAATAAGCTGATTTAACTTCTGTTTTATTTTTATTTATTTTTATCTTTATCT (660)
IR64       CTAGACAATAATAAGCTGATTTAACTTCTGTTTTATTTTTATTTATTTTTATCTTTATCT (660)
AZ1711     CTAGACAATAATAAGCTGATTTAACTTCTGTTTTATTTTTATTTATTTTTATCTTTATCT (660)
           ************************************************************

Nipponbare TTTTTAACAAATTTGTAATTTGAGCTGTGAAATCATAGCTTACTGCCGTTTTGATCGATC (717)
9311       TTTTTAACAAATTTGTAATTTGAGCTGTGAAATCATAGCTTACTGCCGTTTTGATCGATC (720)
IR64       TTTTTAACAAATTTGTAATTTGAGCTGTGAAATCATAGCTTACTGCCGTTTTGATCGATC (720)
AZ1711     TTTTTAACAAATTTGTAATTTGAGCTGTGAAATCATAGCTTACTGCCGTTTTGATCGATC (720)
           ************************************************************

                               ─────────── R3 residues ───────────
Nipponbare GTGTATATATGTTGTCAGGTGGTCTCTCATTGCAGGCAGGCTGCCGGGCCGAACAGACAA (777)
9311       GTGTATATATGTTGTCAGGTGGTCTCTCATTGCAGGCAGGCTGCCGGGCCGAACAGACAA (780)
IR64       GTGTATATATGTTGTCAGGTGGTCTCTCATTGCAGGCAGGCTGCCGGGCCGAACAGACAA (780)
AZ1711     GTGTATATATGTTGTCAGGTGGTCTCTCATTGCAGGCAGGCTGCCGGGCCGAACAGACAA (780)
           ************************************************************
Nipponbare                       S  L  I  A  G  R  L  P  G  R  T  D  N  (101)
9311                             S  L  I  A  G  R  L  P  G  R  T  D  N  (102)
IR64                             S  L  I  A  G  R  L  P  G  R  T  D  N  (102)
AZ1711                           S  L  I  A  G  R  L  P  G  R  T  D  N  (102)
```

```
Nipponbare TGAAATCAAGAACTACTGGAACAGCACGCTCAGCCGCAAGATCGGCACCGCCGCCACCGC (837)
9311       TGAAATCAAGAACT----------CACGCTCAGCCGCAAGATCGGCACCGCCGCCACCGC (830)
IR64       TGAAATCAAGAACT----------CACGCTCAGCCGCAAGATCGGCACCGCCGCCACCGC (830)
AZ1711     TGAAATCAAGAACTACTGGAACAGCACGCTCAGCCGCAAGATCGGCACCGCCGCCACCGC (840)
           **************          ************************************
Nipponbare  E  I  K  N  Y  W  N  S  T  L  S  R  K  I  G  T  A  A  T  A  (121)
9311        E  I  K  N  S  -  -  -  R  S  A  A  R  S  A  P  P  P  P  P  (119)
IR64        E  I  K  N  S  -  -  -  R  S  A  A  R  S  A  P  P  P  P  P  (119)
AZ1711      E  I  K  N  Y  W  N  S  T  L  S  R  K  I  G  T  A  A  T  A  (122)

Nipponbare CGCCGCCGGCAGCCGCGGTGGCAGCACGCCGGACACCGCCAGAGCGACGGACGCGGCGTC (897)
9311       CGCCGCCGGCAGCCGCGGTGGCAGCACGCCGGACACCGCCAGAGCGACGGACGCGGCGTC (890)
IR64       CGCCGCCGGCAGCCGCGGTGGCAGCACGCCGGACACCGCCAGAGCGACGGACGCGGCGTC (890)
AZ1711     CGCTGCCGGCAGCCGCGGTGGCAGCACGCCGGACACCGCCAGAGCGACGGACGCGGCGTC (900)
           *** ********************************************************
Nipponbare  A  A  G  S  R  G  G  S  T  P  D  T  A  R  A  T  D  A  A  S  (141)
9311         P  P  A  A  A  V  A  A  R  R  T  P  P  E  R  R  T  R  R  R (139)
IR64         P  P  A  A  A  V  A  A  R  R  T  P  P  E  R  R  T  R  R  R (139)
AZ1711      A  A  G  S  R  G  G  S  T  P  D  T  A  R  A  T  D  A  A  S  (142)

Nipponbare GTCCAGCTCCGTCGTGCCGCCGGGCCAGCAGCAGCAGCCAGCCTCCCGCGCCGACACCGA (957)
9311       GTCCAGCTCCGTCGTGCCGCCGGGCCAGCAGCAGCAGCCAGCCTCCCGCGCCGACACCGA (950)
IR64       GTCCAGCTCCGTCGTGCCGCCGGGCCAGCAGCAGCAGCCAGCCTCCCGCGCCGACACCGA (950)
AZ1711     GTCCAGCTCCGTCGTGCCGCCGGGCCAGCAGCAGCAGCCAGCCTCCCGCGCCGACACCGA (960)
           ************************************************************
Nipponbare  S  S  S  V  V  P  P  G  Q  Q  Q  Q  P  A  S  R  A  D  T  D  (161)
9311         P  A  P  S  C  R  R  A  S  S  S  S  Q  P  P  A  P  T  P  T (159)
IR64         P  A  P  S  C  R  R  A  S  S  S  S  Q  P  P  A  P  T  P  T (159)
AZ1711      S  S  S  V  V  P  P  G  Q  Q  Q  Q  P  A  S  R  A  D  T  D  (162)

Nipponbare CACAGCAACGGCAGCGGCGGCGGCGGCGGCGACGACGACCACCGTGTGGGCGCCCAAGGC (1017)
9311       CACAGCAACGGCAGCGGCGGCGGCGGCGGCGACGACGACCACCGTGTGGGCGCCCAAGGC (1010)
IR64       CACAGCAACGGCAGCGGCGGCGGCGGCGGCGACGACGACCACCGTGTGGGCGCCCAAGGC (1010)
AZ1711     CACAGCAACGGCAGCGGCGGCGGCGGCGGCGACGACGACCACCGTGTGGGCGCCCAAGGC (1020)
           ************************************************************
Nipponbare  T  A  T  A  A  A  A  A  A  A  T  T  T  T  V  W  A  P  K  A  (181)
9311         Q  Q  R  Q  R  R  R  R  R  R  R  R  P  P  C  G  R  P  R  P (179)
IR64         Q  Q  R  Q  R  R  R  R  R  R  R  R  P  P  C  G  R  P  R  P (179)
AZ1711      T  A  T  A  A  A  A  A  A  A  T  T  T  T  V  W  A  P  K  A  (182)

Nipponbare CGTGCGGTGCACGCGCGGGTTCTTCTTCCACGACCGTGAAACGGCGCCGCTCGCCGCGGC (1077)
9311       CGTGCGGTGCACGCGCGGGTTCTTCTTCCACGACCGTGAAACGGCGCCGCTCGCCGCGGC (1070)
IR64       CGTGCGGTGCACGCGCGGGTTCTTCTTCCACGACCGTGAAACGGCGCCGCTCGCCGCGGC (1070)
AZ1711     CGTGCGGTGCACGCGCGGGTTCTTCTTCCACGACCGTGAAACGGCGCCGCTCGCCGCGGC (1080)
           ************************************************************
Nipponbare  V  R  C  T  R  G  F  F  F  H  D  R  E  T  A  P  L  A  A  A  (201)
9311         C  G  A  R  A  G  S  S  S  T  T  V  K  R  R  R  S  P  R  R (199)
IR64         C  G  A  R  A  G  S  S  S  T  T  V  K  R  R  R  S  P  R  R (199)
AZ1711      V  R  C  T  R  G  F  F  F  H  D  R  E  T  A  P  L  A  A  A  (202)
```

```
Nipponbare GGCGCCGGCGCCGGCAGGGGAATTAGGAGACGGCGATGACGTCGACTGCGACTACTACTG (1137)
9311       GGCGCCGGCGCCGGCAGGGGAATTAGGAGACGGCGATGACGTCGACTGCGACTACTACTG (1130)
IR64       GGCGCCGGCGCCGGCAGGGGAATTAGGAGACGGCGATGACGTCGACTGCGACTACTACTG (1130)
AZ1711     GGCGCCGGCGCCGGCAGGGGAATTAGGAGACGGCGATGACGTCGACTGCGACTACTACTG (1140)
           ************************************************************
Nipponbare  A  P  A  P  A  G  E  L  G  D  G  D  D  V  D  C  D  Y  Y  C  (221)
9311         R  R  R  R  Q  G  N  *                                     (206)
IR64         R  R  R  R  Q  G  N  *                                     (206)
AZ1711      A  P  A  P  A  G  E  L  G  D  G  D  D  V  D  C  D  Y  Y  C  (222)

Nipponbare CAGCGGCAGCAGCTCGGCGGCGACGACGACGTCGTCGAGCTCATTACCGGCGGTCGTCGA (1197)
9311       CAGCGGCAGCAGCTCGGCGGCGACGACGACGTCGTCGAGCTCATTACCGGCGGTCGTCGA (1190)
IR64       CAGCGGCAGCAGCTCGGCGGCGACGACGACGTCGTCGAGCTCATTACCGGCGGTCGTCGA (1190)
AZ1711     CAGCGGCAGCAGCTCGGCGGCGACGACGACGTCGTCGAGCTCATTACCGGTGGTCGTCGA (1200)
           ************************************************** *********
Nipponbare  S  G  S  S  S  A  A  T  T  T  S  S  S  S  L  P  A  V  V  E  (241)
AZ1711      S  G  S  S  S  A  A  T  T  T  S  S  S  S  L  P  V  V  V  E  (242)

Nipponbare GCCGTGCTTCTCCGCCGGCGACGACTGGATGGACGACGTGAGAGCCTTGGCGTCGTTTCT (1257)
9311       GCCGTGCTTCTCCGCCGGCGACGACTGGATGGACGACGTGAGAGCCTTGGCGTCGTTTCT (1250)
IR64       GCCGTGCTTCTCCGCCGGCGACGACTGGATGGACGACGTGAGAGCCTTGGCGTCGTTTCT (1250)
AZ1711     GCCGTGCTTCTCCGCCGGCGACGACTGGATGGACGACGTGAGAGCCTTGGCGTCGTTTCT (1260)
           ************************************************************
Nipponbare  P  C  F  S  A  G  D  D  W  M  D  D  V  R  A  L  A  S  F  L  (261)
AZ1711      P  C  F  S  A  G  D  D  W  M  D  D  V  R  A  L  A  S  F  L  (262)

Nipponbare TGACACCGACGACGCCTGGAACTTGTGTGCGTGA                           (1291)
9311       TGACACCGACGACGCCTGGAACTTGTGTGCGTGA                           (1284)
IR64       TGACACCGACGACGCCTGGAACTTGTGTGCGTGA                           (1284)
AZ1711     TGACACCGACGACGCCTGGAACTTGTGTGCGTGA                           (1294)
           **********************************
Nipponbare  D  T  D  D  A  W  N  L  C  A  *                                 (271)
AZ1711      D  T  D  D  A  W  N  L  C  A  *                                 (272)
```

Figure S3. Nucleotide and amino acid sequence of *OsC1* gene

```
Nipponbare ATGGACACCGGCCGGTCCAAATCCTCGTCGAGGCTGCACCTTGTGATCTTCCCATGGCTC (60)
9311       ATGGGCACCGGCCGGTCCAAATCCTCGTCGAGGCTGCACCTTGTGATCTTCCCATGGCTC
IR64       ATGGACACCGGCCGGTCCAAATCCTCGTCGAGGCTGCACCTTGTGATCTTCCCATGGCTC
AZ1711     ATGGGCACCGGCCGGTCCAAATCCTCGTCGAGGCTGCACCTTGTGATCTTCCCATGGCTC
           **** *******************************************************
Nipponbare M  D  T  G  R  S  K  S  S  S  R  L  H  L  V  I  F  P  W  L   (20)
9311       M  G  T  G  R  S  K  S  S  S  R  L  H  L  V  I  F  P  W  L
IR64       M  D  T  G  R  S  K  S  S  S  R  L  H  L  V  I  F  P  W  L
AZ1711     M  G  T  G  R  S  K  S  S  S  R  L  H  L  V  I  F  P  W  L

Nipponbare GCGTTCGGCCACCTGCTCCCTTACCTCGAGCTCGCGGAGCGCGTGGCGTCGCGTGGCCAC (120)
9311       GCGTTCGGCCACCTGCTCCCTTACCTCGAGCTCGCGGAGCGCGTGGCGTCGCGTGGCCAC
IR64       GCGTTCGGCCACCTGCTCCCTTACCTCGAGCTCGCGGAGCGCGTGGCGTCGCGTGGCCAC
AZ1711     GCGTTCGGCCACCTGCTCCCTTACCTCGAGCTCGCGGAGCGCGTGGCGTCGCGTGGCCAC
           ************************************************************
Nipponbare A  F  G  H  L  L  P  Y  L  E  L  A  E  R  V  A  S  R  G  H   (40)
9311       A  F  G  H  L  L  P  Y  L  E  L  A  E  R  V  A  S  R  G  H
IR64       A  F  G  H  L  L  P  Y  L  E  L  A  E  R  V  A  S  R  G  H
AZ1711     A  F  G  H  L  L  P  Y  L  E  L  A  E  R  V  A  S  R  G  H

Nipponbare CGCGTGTCCTTCGTCTCCACGCCACGCAACCTCGCGCGCCTCCCG--------------- (165)
9311       CGCGTGTCCTTCGTCTCCACGCCACGCAACCTCGCGCGCCTCCCGCCGGTGAGCCCCGCC (180)
IR64       CGCGTGTCCTTCGTCTCCACGCCACGCAACCTCGCGCGCCTCCCG--------------- (165)
AZ1711     CGCGTGTCCTTCGTCTCCACGCCACGCAACCTCGCGCGCCTCCCGCCGGTGAGCCCCGCC (180)
           *********************************************
Nipponbare R  V  S  F  V  S  T  P  R  N  L  A  R  L  P  -  -  -  -  -   (55)
9311       R  V  S  F  V  S  T  P  R  N  L  A  R  L  P  P  V  S  P  A   (60)
IR64       R  V  S  F  V  S  T  P  R  N  L  A  R  L  P  -  -  -  -  -   (55)
AZ1711     R  V  S  F  V  S  T  P  R  N  L  A  R  L  P  P  V  S  P  A   (60)

Nipponbare ---------------------------------------------------------GAC (168)
9311       GCGGCGACGCGCGTCGACCTCGTGGCGCTGCCGCTCCCGCGCGTTCAGGGCCTCCCGGAC (240)
IR64       ---------------------------------------------------------GAC (168)
AZ1711     GCGGCGACGCGCGTCGACCTCGTGGCGCTGCCGCTCCCGCGCGTTCAGGGCCTCCCGGAC (240)
                                                                    ***
Nipponbare -  -  -  -  -  -  -  -  -  -  -  -  -  -  -  -  -  -  -  D   (56)
9311       A  A  T  R  V  D  L  V  A  L  P  L  P  R  V  Q  G  L  P  D   (80)
IR64       -  -  -  -  -  -  -  -  -  -  -  -  -  -  -  -  -  -  -  D   (56)
AZ1711     A  A  T  R  V  D  L  V  A  L  P  L  P  R  V  Q  G  L  P  D   (80)

Nipponbare GGCACCGAGTGCACCAACGACGTTCCCTCCGGCAAGTTCGAGCTCCTCTGGAAGGCCTTC (228)
9311       GGCACCGAGTGCACCAACGACGTTCCCTCCGGCAAGTTCGAGCTCCTCTGGAAGGCCTTC (300)
IR64       GGCACCGAGTGCACCAACGACGTTCCCTCCGGCAAGTTCGAGCTCCTCTGGAAGGCCTTC (228)
AZ1711     GGCACCGAGTGCACCAACGACGTTCCCTCCGGCAAGTTCGAGCTCCTCTGGAAGGCCTTC (300)
           ************************************************************
Nipponbare G  T  E  C  T  N  D  V  P  S  G  K  F  E  L  L  W  K  A  F   (76)
9311       G  T  E  C  T  N  D  V  P  S  G  K  F  E  L  L  W  K  A  F   (100)
IR64       G  T  E  C  T  N  D  V  P  S  G  K  F  E  L  L  W  K  A  F   (76)
AZ1711     G  T  E  C  T  N  D  V  P  S  G  K  F  E  L  L  W  K  A  F   (100)
```

```
Nipponbare GACGCCCTCGCCGCGCCGTTCGCGGAGTTCCTGGGCGCCGCGTGCGACGCCGCCGGCGAG (288)
9311       GACGCCCTCGCCGCGCCGTTCGCGGAGTTCCTGGGCGCCGCGTGCGACGCCGCCGGCGAG (360)
IR64       GACGCCCTCGCCGCGCCGTTCGCGGAGTTCCTGGGCGCCGCGTGCGACGCCGCCGGCGAG (288)
AZ1711     GACGCCCTCGCCGCGCCGTTCGCGGAGTTCCTGGGCGCCGCGTGCGACGCCGCCGGCGAG (360)
           ************************************************************
Nipponbare D  A  L  A  A  P  F  A  E  F  L  G  A  A  C  D  A  A  G  E   (96)
9311       D  A  L  A  A  P  F  A  E  F  L  G  A  A  C  D  A  A  G  E   (120)
IR64       D  A  L  A  A  P  F  A  E  F  L  G  A  A  C  D  A  A  G  E   (96)
AZ1711     D  A  L  A  A  P  F  A  E  F  L  G  A  A  C  D  A  A  G  E   (120)

Nipponbare AGGCCTGATTGGATCATCGCCGACACGTTCCACCACTGGGCACCCCTGGTCGCCCTCCAG (348)
9311       AGGCCTGATTGGATCATCGCCGACACGTTCCACCACTGGGCGCCCCTCGTCGCCCTCCAG (420)
IR64       AGGCCTGATTGGATCATCGCCGACACGTTCCACCACTGGGCACCCCTGGTCGCCCTCCAG (348)
42303      AGGCCTGATTGGATCATCGCCGACACGTTCCACCACTGGGCGCCCCTCGTCGCCCTCCAG (420)
           **************************************** ***** *************
Nipponbare R  P  D  W  I  I  A  D  T  F  H  H  W  A  P  L  V  A  L  Q   (116)
9311       R  P  D  W  I  I  A  D  T  F  H  H  W  A  P  L  V  A  L  Q   (140)
IR64       R  P  D  W  I  I  A  D  T  F  H  H  W  A  P  L  V  A  L  Q   (116)
AZ1711     R  P  D  W  I  I  A  D  T  F  H  H  W  A  P  L  V  A  L  Q   (140)

Nipponbare CACAAGGTGCCATGCGCGATGCTTCTCCCGAGCGCCTCCATGATGGCCGGATGGGCCACC (408)
9311       CACAAGGTGCCATGCGCGATGCTTCTCCCGAGCGCCTCCATGATGGCCGGATGGGCCACC (480)
IR64       CACAAGGTGCCATGCGCGATGCTTCTCCCGAGCGCCTCCATGATGGCCGGATGGGCCACC (408)
AZ1711     CACAAGGTGCCATGCGCGATGCTTCTCCCGAGCGCCTCCATGATGGCCGGATGGGCCACC (480)
           ************************************************************
Nipponbare H  K  V  P  C  A  M  L  L  P  S  A  S  M  M  A  G  W  A  T   (136)
9311       H  K  V  P  C  A  M  L  L  P  S  A  S  M  M  A  G  W  A  T   (160)
IR64       H  K  V  P  C  A  M  L  L  P  S  A  S  M  M  A  G  W  A  T   (136)
AZ1711     H  K  V  P  C  A  M  L  L  P  S  A  S  M  M  A  G  W  A  T   (160)

Nipponbare CGGTCGTCGGAGCCAGCCGGCGCTTCCATCTTCCAAGTGCTCGGCGTGGTAGAAGAACGC (468)
9311       CGGTCGTCGGAGCCAGCCGGCGCTTCAATCTTCCATGTGCTCGGCGCGGTAGAAGAACGC (540)
IR64       CGGTCGTCGGAGCCAGCCGGCGCTTCCATCTTCCAAGTGCTCGGCGTGGTAGAAGAACGC (468)
AZ1711     CGGTCGTCGGAGCCAGCCGGCGCTTCAATCTTCCATGTGCTCGGCGCGGTAGAAGAACGC (540)
           ************************** ******** ********** *************
Nipponbare R  S  S  E  P  A  G  A  S  I  F  Q  V  L  G  V  -  -  -  -   (152)
9311       R  S  S  E  P  A  G  A  S  I  F  H  V  L  G  A  V  E  E  R   (180)
IR64       R  S  S  E  P  A  G  A  S  I  F  Q  V  L  G  V  V  E  E  R   (156)
AZ1711     R  S  S  E  P  A  G  A  S  I  F  H  V  L  G  A  V  E  E  R   (180)

Nipponbare CGGGAGGGCGTGCCATGTTAAGAGTGGGCGCAGAAGGCAAGCTTCTTCGTCGACCATGGC (528)
9311       CGGGAGGGCGTGCCACGTTACGAGTGGGGGCAGAAGGCAAGATTCTTCATCGACCATGGC (600)
IR64       CGGGAGGGCGTGCCATGTTAAGAGTGGGCGCAGAAGGCAAGCTTCTTCGTCGACCATGGC (528)
AZ1711     CGGGAGGGCGTGCCACGTTACGAGTGGGGGCAGAAGGCAAGATTCTTCATCGACCATGGC (600)
           *************** **** ******* ************ ****** ***********
Nipponbare -  -  -  -  -  -  -  -  W  A  Q  K  A  S  F  F  V  D  H  G   (164)
9311       R  E  G  V  P  R  Y  E  W  G  Q  K  A  R  F  F  I  D  H  G   (200)
IR64       R  E  G  V  P  C  *                                          (162)
AZ1711     R  E  G  V  P  R  Y  E  W  G  Q  K  A  R  F  F  I  D  H  G   (200)
```

```
Nipponbare GCGTCGGGCATGTCCGTCGCGAAGCGCTGCTCCTTGGCGATGGAGAGGTGCACGCTGGCG (588)
9311       GCGTCGGGCATGTCCGTCGCGAAGCGCTGCTCCTTGGCGATGGAGAGGTGCACGCTGGCG (660)
IR64       GCGTCGGGCATGTCCGTCGCGAAGCGCTGCTCCTTGGCGATGGAGAGGTGCACGCTGGCG (588)
AZ1711     GCGTCGGGCATGTCCGTCGCGAAGCGCTGCTCCTTGGCGATGGAGAGGTGCACGCTGGCG (660)
           ************************************************************
Nipponbare A  S  G  M  S  V  A  K  R  C  S  L  A  M  E  R  C  T  L  A   (184)
9311       A  S  G  M  S  V  A  K  R  C  S  L  A  M  E  R  C  T  L  A   (220)
IR64
AZ1711     A  S  G  M  S  V  A  K  R  C  S  L  A  M  E  R  C  T  L  A   (220)

Nipponbare GCCATGCGGAGCTGCCCGGAGTGGGAGCCCGACGCCTTCCAGCAGGTGGCGGCGGGCCTG (648)
9311       GCCATGCGGAGCTGCCCGGAGTGGGAGCCCGACGCCTTCCAGCAGGTGGCGGCGGGCCTG (720)
IR64       GCCATGCGGAGCTGCCCGGAGTGGGAGCCCGACGCCTTCCAGCAGGTGGCGGCGGGCCTG (648)
AZ1711     GCCATGCGGAGCTGCCCGGAGTGGGAGCCCGACGCCTTCCAGCAGGTGGCGGCGGGCCTG (720)
           ************************************************************
Nipponbare A  M  R  S  C  P  E  W  E  P  D  A  F  Q  Q  V  A  A  G  L   (204)
9311       A  M  R  S  C  P  E  W  E  P  D  A  F  Q  Q  V  A  A  G  L   (240)
IR64
AZ1711     A  M  R  S  C  P  E  W  E  P  D  A  F  Q  Q  V  A  A  G  L   (240)

Nipponbare AAGAACAAGCCGCTCATCCCCCTCGGCCTCGTGCCACCGTCGCCCGACGGAGGCCGCCGC (708)
9311       AAGAACAAGCCGCTCATCCCCCTCGGCCTCGTGCCACCGTCGCCCGACGGAGGCCGCCGC (780)
IR64       AAGAACAAGCCGCTCATCCCCCTCGGCCTCGTGCCACCGTCGCCCGACGGAGGCCGCCGC (708)
AZ1711     AAGAACAAGCCGCTCATCCCCCTCGGCCTCGTGCCACCGTCGCCCGACGGAGGCCGCCGC (780)
           ************************************************************
Nipponbare K  N  K  P  L  I  P  L  G  L  V  P  P  S  P  D  G  G  R  R   (224)
9311       K  N  K  P  L  I  P  L  G  L  V  P  P  S  P  D  G  G  R  R   (260)
IR64
AZ1711     K  N  K  P  L  I  P  L  G  L  V  P  P  S  P  D  G  G  R  R   (260)

Nipponbare CGCGCCGGCATGACGGACAACTCGACGATGCGGTGGCTGGACGTGCAGCCGGCGAAGTCG (768)
9311       CGTGCCGGCATGACGGACAACTCGACGATGCGGTGGCTGGACGTGCAGCCGGCGAAGTCG (840)
IR64       CGCGCCGGCATGACGGACAACTCGACGATGCGGTGGCTGGACGTGCAGCCGGCGAAGTCG (768)
AZ1711     CGTGCCGGCATGACGGACAACTCGACGATGCGGTGGCTGGACGTGCAGCCGGCGAAGTCG (840)
           ** *********************************************************
Nipponbare R  A  G  M  T  D  N  S  T  M  R  W  L  D  V  Q  P  A  K  S   (244)
9311       R  A  G  M  T  D  N  S  T  M  R  W  L  D  V  Q  P  A  K  S   (280)
IR64
AZ1711     R  A  G  M  T  D  N  S  T  M  R  W  L  D  V  Q  P  A  K  S   (280)

Nipponbare GTGGTGTACGTGGCGCTGGGGAGCGAGGTGCCTCTACCATTGGAGCAGGTGCACGAGCTG (828)
9311       GTGGTGTACGTGGCGCTGGGCAGCGAGGTGCCTCTACCATTGGAGCAGGTGCACGAGCTG (900)
IR64       GTGGTGTACGTGGCGCTGGGGAGCGAGGTGCCTCTACCATTGGAGCAGGTGCACGAGCTG (828)
AZ1711     GTGGTGTACGTGGCGCTGGGCAGCGAGGTGCCTCTACCATTGGAGCAGGTGCACGAGCTG (900)
           ******************** ***************************************
Nipponbare V  V  Y  V  A  L  G  S  E  V  P  L  P  L  E  Q  V  H  E  L   (264)
9311       V  V  Y  V  A  L  G  S  E  V  P  L  P  L  E  Q  V  H  E  L   (300)
IR64
AZ1711     V  V  Y  V  A  L  G  S  E  V  P  L  P  L  E  Q  V  H  E  L   (300)
```

```
Nipponbare GCTCTCGGGCTGGAGCTCGCCGGGACACGATTTCTGTGGGCGCTGAGAAAGCCCCATGGC (888)
9311       GCTCTCGGGCTGGAGCTCGCCGGGACACGGTTTCTGTGGGCGCTGAGAAAGCCCCATGGC (960)
IR64       GCTCTCGGGCTGGAGCTCGCCGGGACACGATTTCTGTGGGCGCTGAGAAAGCCCCATGGC (888)
AZ1711     GCTCTCGGGCTGGAGCTCGCCGGGACACGGTTTCTGTGGGCGCTGAGAAAGCCCCATGGC (960)
           ***************************** ******************************
Nipponbare A  L  G  L  E  L  A  G  T  R  F  L  W  A  L  R  K  P  H  G    (284)
9311       A  L  G  L  E  L  A  G  T  R  F  L  W  A  L  R  K  P  H  G    (320)
IR64
AZ1711     A  L  G  L  E  L  A  G  T  R  F  L  W  A  L  R  K  P  H  G    (320)

Nipponbare GTCGATCTCTCAGATGTCCTTCCTCCGGGTTACCAAGAACGGACGAAGAGCCATGGACAT (948)
9311       GTCGATCTCTCAGATGTCCTTCCTCCGGGTTACCAAGAACGGACGAAGAGCCATGGACAT (1020)
IR64       GTCGATCTCTCAGATGTCCTTCCTCCGGGTTACCAAGAACGGACGAAGAGCCATGGACAT (948)
AZ1711     GTCGATCTCTCAGATGTCCTTCCTCCGGGTTACCAAGAACGGACGAAGAGCCATGGACAT (1020)
           ************************************************************
Nipponbare V  D  L  S  D  V  L  P  P  G  Y  Q  E  R  T  K  S  H  G  H    (304)
9311       V  D  L  S  D  V  L  P  P  G  Y  Q  E  R  T  K  S  H  G  H    (340)
IR64
AZ1711     V  D  L  S  D  V  L  P  P  G  Y  Q  E  R  T  K  S  H  G  H    (340)

Nipponbare GTTGCCATGGGGTGGGTTCCGCAGATCACCATACTGGCGCACGCCGCGGTGGGCGCCTTC (1008)
9311       GTTGCCATGGGGTGGGTTCCGCAGATCACCATACTGGCGCACGCGGCGGTGGGCGCCTTC (1080)
IR64       GTTGCCATGGGGTGGGTTCCGCAGATCACCATACTGGCGCACGCCGCGGTGGGCGCCTTC (1008)
AZ1711     GTTGCCATGGGGTGGGTTCCGCAGATCACCATACTGGCGCACGCGGCGGTGGGCGCCTTC (1080)
           ******************************************** ***************
Nipponbare V  A  M  G  W  V  P  Q  I  T  I  L  A  H  A  A  V  G  A  F    (324)
9311       V  A  M  G  W  V  P  Q  I  T  I  L  A  H  A  A  V  G  A  F    (360)
IR64
AZ1711     V  A  M  G  W  V  P  Q  I  T  I  L  A  H  A  A  V  G  A  F    (360)

Nipponbare TTGACGCACTGTGGCCGCAACTCGCTCGTCGAGGGGCTTCTGTTTGGGAACCCTCTCATC (1068)
9311       TTGACGCACTGTGGCCGCAACTCGCTCGTAGAGGGGCTTCTGTTTGGGAACCCTCTCGTC (1140)
IR64       TTGACGCACTGTGGCCGCAACTCGCTCGTCGAGGGGCTTCTGTTTGGGAACCCTCTCATC (1068)
AZ1711     TTGACGCACTGTGGCCGCAACTCGCTCGTAGAGGGGCTTCTGTTTGGGAACCCTCTCGTC (1140)
           ***************************** *************************** **
Nipponbare L  T  H  C  G  R  N  S  L  V  E  G  L  L  F  G  N  P  L  I    (344)
9311       L  T  H  C  G  R  N  S  L  V  E  G  L  L  F  G  N  P  L  I    (380)
IR64
AZ1711     L  T  H  C  G  R  N  S  L  V  E  G  L  L  F  G  N  P  L  I    (380)

Nipponbare ATGTTGCCCATCTTCGGGGACCAAGGGCCGAACGCGCGGCTCATGGAGGGTAACAAGGTA (1128)
9311       ATGTTGCCCATCTTCGGGGACCAAGGGCCGAACGCGCGGCTCATGGAGAGTAACAAGGTA (1200)
IR64       ATGTTGCCCATCTTCGGGGACCAAGGGCCGAACGCGCGGCTCATGGAGGGTAACAAGGTA (1128)
AZ1711     ATGTTGCCCATCTTCGGGGACCAAGGGCCGAACGCGCGGCTCATGGAGAGTAACAAGGTA (1200)
           ************************************************ ***********
Nipponbare M  L  P  I  F  G  D  Q  G  P  N  A  R  L  M  E  G  N  K  V    (364)
9311       M  L  P  I  F  G  D  Q  G  P  N  A  R  L  M  E  G  N  K  V    (400)
IR64
AZ1711     M  L  P  I  F  G  D  Q  G  P  N  A  R  L  M  E  G  N  K  V    (400)
```

```
Nipponbare GGATCGCAGGTGAGGCGGGATGACATGGACGGGTCATTCGACCGCCATGGCGTCGCCGCC (1188)
9311       GGATCGCAGGTGAGGCGGGATGACATGGACGGGTCATTCGACCGCCATGGCGTCGCCGCC (1260)
IR64       GGATCGCAGGTGAGGCGGGATGACATGGACGGGTCATTCGACCGCCATGGCGTCGCCGCC (1188)
AZ1711     GGATCGCAGGTGAGGCGGGATGACATGGACGGGTCATTCGACCGCCATGGCGTCGCCGCC (1260)
           ************************************************************
Nipponbare G  S  Q  V  R  R  D  D  M  D  G  S  F  D  R  H  G  V  A  A   (384)
9311       G  S  Q  V  R  R  D  D  M  D  G  S  F  D  R  H  G  V  A  A   (420)
IR64
AZ1711     G  S  Q  V  R  R  D  D  M  D  G  S  F  D  R  H  G  V  A  A   (420)

Nipponbare GCGGTCCGGGCCGTCATGGTTGAGGAAGAAACCAGGAGGGTGTTTGTAGCAAACGCCATC (1248)
9311       GCGGTCCGGGCCGTCATGGTTGAGGAAGAAACCAGGAGGGTGTTTGTAGCAAACGCCATC (1320)
IR64       GCGGTCCGGGCCGTCATGGTTGAGGAAGAAACCAGGAGGGTGTTTGTAGCAAACGCCATC (1248)
AZ1711     GCGGTCCGGGCCGTCATGGTTGAGGAAGAAACCAGGAGGGTGTTTGTAGCAAACGCCATC (1320)
           ************************************************************
Nipponbare A  V  R  A  V  M  V  E  E  E  T  R  R  V  F  V  A  N  A  I   (404)
9311       A  V  R  A  V  M  V  E  E  E  T  R  R  V  F  V  A  N  A  I   (440)
IR64
AZ1711     A  V  R  A  V  M  V  E  E  E  T  R  R  V  F  V  A  N  A  I   (440)

Nipponbare AGGCTGCAAGAGCTTGTAGCTGACAAGGAACTCCATGAGAGGTACATTGACGAGTTTATA (1308)
9311       AGGCTGCAAGAGATTGTAGTTGACAAGGAACTCCATGGGAGGTACATCGACGAGTTTATA (1380)
IR64       AGGCTGCAAGAGCTTGTAGCTGACAAGGAACTCCATGAGAGGTACATTGACGAGTTTATA (1308)
AZ1711     AGGCTGCAAGAGATTGTAGTTGACAAGGAACTCCATGGGAGGTACATCGACGAGTTTATA (1380)
           ************ ****** ***************** ********* ************
Nipponbare R  L  Q  E  L  V  A  D  K  E  L  H  E  R  Y  I  D  E  F  I   (424)
9311       R  L  Q  E  L  V  A  D  K  E  L  H  E  R  Y  I  D  E  F  I   (460)
IR64
AZ1711     R  L  Q  E  L  V  A  D  K  E  L  H  E  R  Y  I  D  E  F  I   (460)

Nipponbare CAACAGCTTGTTTCTCACGGCGCGGATGGAAGCTGCAATACTGCCGCCCCAGTTCCTTCC (1368)
9311       CAACAGCTTGTTTCTCAGGGCGCGGATGGAAGCTGCAATACTGCCGCCCTAGTTCCTTCC (1440)
IR64       CAACAGCTTGTTTCTCACGGCGCGGATGGAAGCTGCAATACTGCCGCCCCAGTTCCTTCC (1368)
AZ1711     CAACAGCTTGTTTCTCAGGGCGCGGATGGAAGCTGCAATACTGCCGCCCTAGTTCCTTCC (1440)
           ***************** ******************************* **********
Nipponbare Q  Q  L  V  S  H  G  A  D  G  S  C  N  T  A  A  P  V  P  S   (444)
9311       Q  Q  L  V  S  H  G  A  D  G  S  C  N  T  A  A  P  V  P  S   (480)
IR64
AZ1711     Q  Q  L  V  S  H  G  A  D  G  S  C  N  T  A  A  P  V  P  S   (480)

Nipponbare AGCTAA                                                       (1374)
9311       AGCTAA                                                       (1446)
IR64       AGCTAA                                                       (1374)
AZ1711     AGCTAA                                                       (1446)
           ******
Nipponbare S  *                                                         (445)
9311       S  *                                                         (481)
IR64
AZ1711     S  *                                                         (481)
```

Figure S4. Nucleotide and amino acid sequence of *3GT* gene
