# Supplementary material for: Reverse Mutations in Pigmentation Induced by Sodium Azide in the IR64 Rice Variety
Source: Curr Issues Mol Biol. 2024 Nov 22;46(12):13328–46. doi: 10.3390/cimb46120795 (PMC11727009; doi:10.3390/cimb46120795)
Supplement: Supplementary file 1 [file cimb-46-00795-s001.zip › 20241010 Sup Table for cimb KJ.pdf]

Supporting Information

Article title: Reverse pigmentation mutations induced by sodium azide in the IR64 rice variety

Authors: Hsian-Jun Chen, Anuchart Sawasdee, Yu-Ling Lin, Min-Yu Chiang, Hsin-Yi Chang, Wen-Hsiung Li, Chang-Sheng Wang

Table S1. The SSR markers were applied in polymorphism screening

**Chr**	**Marker name (Bold name = polymorphic marker)**									
**1**	RM462	**RM495**	RM499	RM1331	RM323	RM283	RM272	RM259	**RM579**	**RM493**
	**RM449**	RM5964	**RM5638**	RM8089	RM6880	RM5853	**RM6716**	RM9	RM5	**RM2318**
	RM5919	**RM488**	RM3475	RM237	**RM246**	RM443	RM403	RM543	RM212	**RM1003**
	RM226	RM312	RM486	RM265	RM315	RM431	RM104	RM14	RM568	RM6407
**2**	RM109	RM154	RM110	RM211	RM233a	RM279	**RM3865a**	RM423	RM53	RM555
	RM5897	**RM174**	RM6375	**RM6911**	RM5356	RM452	**RM1313**	RM550	RM438	RM27
	RM300	RM29	**RM6611**	RM341	RM475	RM1694	RM1303	RM5430	RM106	RM6107
	RM526	RM3512	**RM263**	RM3508	RM497	RM221	RM318	RM3316	RM530	RM112
	RM3302	RM425	RM250	RM6030	RM5300	**RM482**	RM498	RM535		
**3**	RM5849	RM4108	RM132	RM569	RM175	RM143	**RM489**	**RM545**	**RM5444**	RM517
	RM3716	RM3872	**RM7**	RM218	**RM232**	**RM251**	RM563	RM282	RM338	RM6594
	**RM156**	RM6881	RM2346	**RM5864**	RM16	RM6266	RM347	RM503	RM135	**RM426**
	**RM168**	RM186	**RM55**	RM3199	RM3525	RM416	RM293	RM1352a	RM6806	RM6970
	RM468	**RM422**	**RM514**	RM570	**RM85**					
**4**	RM335	**RM518**	RM261	RM7472	RM307	RM401	RM7113	RM185	**RM471**	RM417
	RM6997	RM142	RM7563	RM119	RM1388a	RM2521	RM177	RM5320	RM273	RM252
	RM5720	RM241	RM7187	RM451	RM303	RM317	RM6089	RM3276	**RM3319**	RM348
	RM7314	RM131	RM124	RM559	RM6156	RM5900	RM1359a	RM1155	RM3337	RM3866
	RM6005									
**5**	RM507	**RM1248**	RM2010	**RM1024**	**RM413**	**RM13**	**RM437**	RM3328	RM169	RM289
	RM3381	RM509	RM6645	RM598	RM430	RM146	**RM164**	RM459	RM161	RM534
	RM188	RM2357	**RM3870**	RM421	**RM5970**	RM178	RM87	**RM480**	RM334	
**6**	RM133	**RM435**	RM190	RM587	RM510	**RM225**	RM50	RM539	**RM6917**	**RM6773**
	**RM5754**	**RM253**	**RM2615**	**RM276**	**RM549**	**RM136**	**RM527**	RM541	RM7551	RM3827
	RM162	RM275	**RM528**	RM3	RM340	RM400	RM103	RM412	RM6857	RM6119
	RM7561	RM19618	RM19467	RM3805	RM6003	RM6263	RM585	RM19423	RM6057	RM19334
	RM19366	RM19388	RM19399	RM19406	RM19535	RM19548	RM19573	RM19588	RM19596	RM19602
	RM19609	RM469	RM7399	RM597	RM586	RM6536	RM7420	RM111	RM19556	RM19565
	RM6779	RM6701	RM7583	RM6818	RM1161	RM454	RM7579	RM1340	RM5314a	RM5371
	RM6782									
**7**	**RM295**	**RM5055**	RM427	**RM1134**	RM8263	RM125	RM6776	RM1253	**RM481**	RM501
	RM214	RM3670	RM6449	RM500	RM298	RM445	RM11	RM3403a	RM182	RM70
	RM455	**RM5508**	**RM505**	**RM234**	RM478	**RM118**	**RM248**	RM3555	RM1357	RM420
**8**	RM408	**RM337**	RM1959	RM152	**RM1235a**	RM1295	**RM38**	**RM5556**	RM1111	**RM310**
	RM72	RM6008	RM44	RM483	RM331	RM339	RM42	RM350	RM515	RM284
	**RM210**	RM556	**RM149**	RM230	RM6542	RM433	RM458	RM447	RM477	RM5545
	RM4154									
**9**	RM4413	RM316	RM464	RM524	**RM296**	RM321	**RM105**	RM409	RM460	RM434
	**RM3700**	**RM410**	RM108	RM328	RM160	RM215	RM6816	RM1026	RM6797	RM205
**10**	**RM474**	**RM222**	**RM216**	RM1126	RM239	**RM271**	RM467	RM1083	RM184	**RM1375**
	RM269	RM171	**RM3773**	**RM484**	RM147	**RM228**	RM333	**RM590**	RM6016	
**11**	RM286	RM4484	RM6544	RM332	RM167	RM552	RM116	RM441	RM479	**RM202**
	RM536	**RM287**	**RM1355**	**RM229**	RM21	RM206	RM4601	RM5997	RM6499	RM254
	RM6440	RM224	RM144							
**12**	RM415	RM7582	RM558	RM3483a	RM19	RM247	RM3472	RM491	RM3455	RM512
	RM101	RM2529	RM277	**RM519**	RM313	RM309	RM6410	RM5282	RM270	**RM235**
	**RM2197**									

Table S3. Sequence of primers used in this study

Name	5'>3'	PCR Product length (Wildtype/Mutant) (bp)
OsC1-1F	ATCGCTCAGTCTCACACCGCACAG	1361/1371
OsC1-6R	GTCACGCACACAAGTTCCAGGC	
OsC1-4R	GTTGCTGTGTCGGTGTCGGCG	227/237
OsC1-5F	TGTCAGGTGGTCTCTCATTGCAGG	
3Oglu-3F	AAATCGATCGATAGGTAAGACGGGA	1589/1661
3Oglu-4R	GAGACAAAATCCCCTGGCAAATAG	
3Oglu-6R	TGTCGGCGATGATCCAATCAGG	323/395
3Oglu-RT1	CAACTTCGCCATGGGCACC	

Table S4. The appearances of 10 bp insertion and pigmentation in *japonica* and *indica* rice varieties.

Type	Variety	Insertion of 10 bp	Pigmentation	References
indica	868	NO	Colorless	Saitoh et al., 2004
indica	93-11	NO	Colorless	
indica	Aubalam	NO	Colorless	Choudhury et al., 2014
indica	Bahadur	NO	Colorless	Choudhury et al., 2014
indica	Balam	YES	Colored	Choudhury et al., 2014
indica	Bas Beroin	YES	Colored	Choudhury et al., 2014
indica	Bashful	YES	Colorless	Choudhury et al., 2014
indica	Basmati	NO		
indica	Borjahinga	YES	Colorless	Choudhury et al., 2014
indica	Borua Beroin	YES	Colorless	Choudhury et al., 2014
indica	IR36	NO	Colorless	Saitoh et al., 2004
indica	IR8	NO	Colorless	Choudhury et al., 2014
indica	Joya	NO	Colorless	Choudhury et al., 2014
indica	Kakiberoin	NO	Colorless	Choudhury et al., 2014
indica	Kawanglawang	YES	Colored	Choudhury et al., 2014
indica	Lallatoi	NO	Colored	Choudhury et al., 2014
indica	Minghui 63	NO	Colorless	
indica	Moircha	NO	Colorless	Choudhury et al., 2014
indica	Mulahail	NO	Colorless	Choudhury et al., 2014
indica	Pankaj	NO	Colorless	Choudhury et al., 2014
indica	Papue	NO	Colorless	Choudhury et al., 2014
indica	Ranga Borah	NO	Colorless	Choudhury et al., 2014
indica	Ranjit	NO	Colorless	Choudhury et al., 2014
indica	RP Bio-226	NO		
indica	SA7	NO		Saitoh et al., 2004
indica	SA8	NO		Saitoh et al., 2004
indica	SA9	NO		Saitoh et al., 2004
indica	Shuhui498	NO		
indica	Sorpuma	NO	Colorless	Choudhury et al., 2014
indica	Til Bora	YES	Colored	Choudhury et al., 2014
indica	ZS97	YES	Colored	
japonica	Arfa	NO	Colorless	Choudhury et al., 2014
japonica	Bherapawa	YES	Colored	Choudhury et al., 2014
japonica	Guaroi	NO	Colorless	Choudhury et al., 2014
japonica	Harinarayan	NO	Colorless	Choudhury et al., 2014
japonica	Hati Hali	YES	Colored	Choudhury et al., 2014
japonica	Joha	YES	Colored	Choudhury et al., 2014
japonica	Lahi	YES	Colorless	Choudhury et al., 2014
japonica	Local Basmati	NO	Colorless	Choudhury et al., 2014
japonica	Mimutim	NO	Colorless	Choudhury et al., 2014
japonica	Nipponbare	YES	Colorless	
japonica	SA1	YES		Saitoh et al., 2004
japonica	SA2	YES		Saitoh et al., 2004
japonica	SA4	NO		Saitoh et al., 2004
japonica	SA5	YES		Saitoh et al., 2004
japonica	T65	YES	Colored	Saitoh et al., 2004
Oriza sativa	SA3	YES		Saitoh et al., 2004
Oryza rufipogon	*O. rufipogon*	YES	Colored	Choudhury et al., 2014
